# Smart nanocarriers and biomaterials for targeted drug delivery in spinal cord injury: molecular mechanisms and translational perspectives

**DOI:** 10.3389/fbioe.2026.1973263

**Published:** 2026-10-15

**Authors:** Wenqing Han, Pengfei Qi, Jiaxin Li, Lu Min, Hongxiang Li, Hongna Yin

**Affiliations:** 1 Heilongjiang University of Chinese Medicine, Harbin, China; 2 The Second Affiliated Hospital of Heilongjiang University of Chinese Medicine, Harbin, China

**Keywords:** drug delivery systems, mechanisms of action, nanocarriers, nerve regeneration, spinal cord injury, targeted therapy

## Abstract

Spinal cord injury (SCI) results in permanent motor, sensory, and autonomic dysfunction below the level of injury; currently, there are no effective treatments to promote neural functional recovery. Inflammatory responses, oxidative stress, apoptosis, excitotoxicity, glial scarring, and impaired axonal regeneration—all triggered by secondary damage—are the core pathological mechanisms hindering functional recovery. Traditional drugs, however, are limited by the blood-spinal cord barrier, short half-lives, and systemic toxic side effects, resulting in poor therapeutic efficacy. This article provides a systematic review of research progress over the past decade on the use of nanocarrier-based therapeutics for the treatment of spinal cord injury and analyzes their therapeutic effects through seven major mechanisms, including the inhibition of inflammation and regulation of macrophage/microglia polarization, antagonism of oxidative stress, regulation of programmed cell death and neuroprotection, as well as the promotion of axonal regeneration, myelin repair, glial scar and extracellular matrix remodeling, and vascular repair and blood-spinal cord barrier reconstruction. It further compares the functional characteristics, advantages, and translational potential of major delivery platforms, including liposomes, polymeric nanoparticles, micelles, inorganic nanomaterials, nanozymes, exosomes, biomimetic carriers, nanohydrogels, and composite scaffolds. Among these, biomimetic carriers and multifunctional nanozymes have shown promising preclinical results in trans-barrier delivery and modulation of the injury microenvironment. However, current research still faces bottlenecks such as insufficient long-term safety validation, low targeting efficiency, significant discrepancies between animal models and clinical pathology, and difficulties in scaling up preparation processes. By integrating the characteristics of nanocarriers with the key pathological mechanisms at different stages of spinal cord injury, this paper provides a mechanism-driven analytical framework for the rational design and clinical translation of nanomedicines. In the future, microenvironment-responsive smart delivery, the integration of nanotechnology with stem cells and neuromodulation strategies, and the establishment of standardized evaluation systems may become key directions for advancing the clinical translation of nanomedicines.

## 1 Introduction

Spinal cord injury (SCI) is a severely debilitating disease of the central nervous system (CNS) caused by external forces such as traffic accidents, falls from height, sports injuries, and violence. It is characterized by partial or complete loss of motor, sensory, and sphincter function below the lesion level, with high disability and mortality rates and a low cure rate (Khan et al., 2020; Li et al., 2025). The global annual incidence ranges from approximately ten to more than sixty new cases per million population, and most patients are young and middle-aged adults of working age, imposing a heavy economic and medical burden on families and society (Khan et al., 2020; Gu et al., 2024). Neurological recovery after SCI is extremely difficult, fundamentally because the intrinsic regenerative capacity of the adult mammalian CNS is weak and damaged axons rarely regenerate spontaneously (Zhang et al., 2025; Conklin et al., 2026); meanwhile, persistent local inflammation, massive accumulation of reactive oxygen species (ROS), disintegrated myelin debris, dense glial scars, and inhibitory extracellular matrix collectively constitute multiple barriers to axonal regeneration (Chen et al., 2023; Huang et al., 2023). Moreover, SCI not only causes tissue destruction at the injured segment but also induces chronic metabolic and transcriptomic alterations in remote structures such as the cerebral cortex through the interruption of ascending and descending pathways, further aggravating neurological dysfunction (Boyko et al., 2021; Sugeno et al., 2021).

Current clinical management of SCI still relies mainly on early surgical decompression/reduction and spinal internal fixation, which aim to relieve spinal cord compression and stabilize the spine but cannot reverse the neural tissue destruction that has already occurred (Li et al., 2025; Gu et al., 2024). High-dose methylprednisolone pulse therapy was once widely used for acute SCI to suppress the secondary inflammatory cascade; however, its narrow therapeutic time window and controversial efficacy, together with the serious adverse effects of high-dose glucocorticoids such as gastrointestinal hemorrhage and infection, have led several guidelines to no longer recommend it as routine treatment (Bin et al., 2017; Li B. et al., 2026; Wang et al., 2023). Neurotrophic factors such as brain-derived neurotrophic factor (BDNF) and hepatocyte growth factor (HGF) have shown pro-regenerative effects in animal experiments, but their short half-life, susceptibility to degradation, and poor penetration across the blood-spinal cord barrier (BSCB) limit clinical translation (Yamane et al., 2019; Xia et al., 2021; Yamane et al., 2018). Stem cell transplantation promotes repair through differentiation-based replacement and paracrine effects, but is hampered by low survival of grafted cells, insufficient directed differentiation efficiency, and tumorigenic risk (Xue W. et al., 2021; Han et al., 2018; Zou et al., 2020). In recent years, emerging therapies such as intradural injection of anti-Nogo-A antibodies have achieved certain progress in clinical trials (Weidner et al., 2025; Farner et al., 2026; Shen et al., 2020), and physical interventions such as spinal cord stimulation and wireless optoelectrical modulation have brought new hope for motor function reconstruction (Zhou et al., 2024; Ji et al., 2025; Lin et al., 2023; Jia et al., 2026); nevertheless, a specific therapy capable of simultaneously modulating multiple pathological mechanisms and achieving coordinated reconstruction of neural structure and function is still lacking.

Conventional pharmacotherapy faces common problems: short drug half-lives requiring repeated administration, severely restricted permeability across the BSCB resulting in insufficient local drug concentration at the lesion, systemic distribution to non-target organs causing toxic side effects, and the inability of a single drug to cover the complex and diverse pathological processes of SCI (Ma et al., 2022; Shen et al., 2021; Gao et al., 2022a). Nanocarrier-based drug delivery systems, owing to their small particle size, large specific surface area, and high modifiability, enable targeted drug delivery, sustained and controlled release, and co-delivery of multiple drugs, offering a new avenue for SCI treatment (Khan et al., 2020; Waris et al., 2022; Gao et al., 2022b). Bibliometric analyses in recent years have shown sustained and rapid growth in publications on nanotechnology in the SCI field, marking it as a research frontier and hotspot (Gu et al., 2024). Nanocarriers can achieve active targeting through surface modification: by conjugating antibodies or peptides, they recognize receptors or molecular markers highly expressed in the injured region, thereby increasing local drug accumulation at the lesion (Fan X. et al., 2024; Kong et al., 2025). Nanocarriers are also capable of conditional release in response to characteristic changes in the injury microenvironment such as ROS, matrix metalloproteinases (MMPs), and pH, prolonging the maintenance of effective drug concentrations (Ma et al., 2022; Zhuang et al., 2025; Liu Y. et al., 2020). In addition, biomimetic nanocarriers such as cell membrane-coated nanoparticles and exosomes combine the low immunogenicity of natural biological membranes with good barrier-crossing ability (An et al., 2022; Kim et al., 2018; Qin et al., 2025), while nanohydrogels and composite scaffolds can fill lesion cavities, provide mechanical support and tissue bridging, and achieve long-term sustained release (Santi et al., 2021; Ju et al., 2022). Given these advantages, nanocarrier-based therapeutics are expected to systematically ameliorate the local microenvironment after SCI and represent an important breakthrough direction for SCI therapy (Khan et al., 2020; Li et al., 2025; Nejati-Koshki et al., 2017). Although previous reviews have addressed individual nanocarrier classes or single mechanisms in SCI, systematic comparison and integrated analysis of multiple carrier classes across multiple mechanistic levels are lacking, making it difficult to comprehensively evaluate the efficacy differences and translational potential of different strategies. On this basis, this review systematically surveys the relevant literature of the past decade and performs a cross-analysis along two dimensions, nanocarrier type and multiple therapeutic mechanisms, in order to provide an integrative reference for the rational design and clinical translation of nanomedicines.

## 2 Pathophysiology of spinal cord injury

### 2.1 Inflammatory response and immune microenvironment imbalance

After SCI, the BSCB is rapidly disrupted, and peripheral neutrophils, monocytes/macrophages, and lymphocytes sequentially infiltrate the lesion, while resident microglia are rapidly activated by injury signals (Ma et al., 2022; An et al., 2022). Classically activated M1 microglia and macrophages release large amounts of pro-inflammatory cytokines such as tumor necrosis factor-alpha (TNF-alpha), interleukin-1beta (IL-1beta), and IL-6, as well as MMPs, further damaging the BSCB and aggravating tissue edema and neuronal death, whereas M2 cells secrete anti-inflammatory factors such as IL-10 and transforming growth factor-beta that facilitate inflammation resolution and tissue repair (An et al., 2022; Zhou et al., 2022; Ma et al., 2020). Imbalanced M1/M2 polarization and chronicization of the inflammatory response after SCI are key drivers of progressive secondary injury; macrophages that excessively phagocytose myelin debris can transform into foam-like cells, markedly aggravating secondary pathological damage (An et al., 2022; Liu Z. et al., 2025). Activation of innate immune signaling platforms such as the nucleotide-binding oligomerization domain-like receptor protein 3 (NLRP3) inflammasome mediates pyroptosis, further amplifying the inflammatory response (M Alsharbaty et al., 2026).

### 2.2 Oxidative stress and multiple forms of cell death

After SCI, impairment of the mitochondrial respiratory chain, calcium overload, and activation of inflammatory cells lead to a burst of reactive oxygen and nitrogen species, whereas the endogenous antioxidant enzyme system is severely decompensated (Li et al., 2019; Andrabi et al., 2020; Lee et al., 2026). Excessive free radicals attack biological macromolecules, causing lipid peroxidation, protein nitration, and oxidative DNA damage, resulting in membrane destruction and death of neurons and glial cells (Andrabi et al., 2020; Singh et al., 2026). Mitochondria are both the main source of ROS and the primary target of oxidative damage; mitochondrial dysfunction and oxidative stress form a vicious cycle (Andrabi et al., 2020; Wei et al., 2025). Recent studies have also found that ferroptosis triggered by iron overload and impaired autophagic flux, among other regulated cell death modalities, play important roles in neuronal loss after SCI; their specific molecular mechanisms are discussed further below in the context of nanocarrier intervention strategies (Feng et al., 2025; Ding et al., 2026; Li RY. et al., 2023). During secondary injury, necrosis predominates at the lesion core, while cells in the surrounding penumbra undergo apoptosis mediated by mitochondrial cytochrome c release and caspase cascade activation (Gao et al., 2022b; Andrabi et al., 2020). Damaged neurons and glial cells release large amounts of excitatory amino acids such as glutamate; excessive activation of NMDA and AMPA receptors causes massive calcium influx, triggering collapse of the mitochondrial membrane potential and excitotoxic neuronal death (Boyko et al., 2018; Boyko et al., 2022).

### 2.3 Glial scar formation and axonal regeneration failure

Reactive astrogliosis is one of the most characteristic pathological changes after SCI. Within days of injury, astrocytes proliferate extensively at the lesion margin and interweave into a dense network, accompanied by infiltration of oligodendrocyte progenitor cells and fibroblasts, collectively forming a glial scar characterized by massive deposition of chondroitin sulfate proteoglycans (CSPGs) (Zhang H. et al., 2021; Sun X. et al., 2026). In the acute phase, the glial scar limits the spread of inflammation and protects residual tissue; in the chronic phase, however, its dense structure, together with inhibitory extracellular matrix molecules, constitutes a physical and chemical barrier to axonal growth, severely impeding axonal regeneration and synaptic reconstruction (Sun X. et al., 2026; Hassanli et al., 2023). Axonal regeneration is further suppressed by myelin-associated inhibitors such as Nogo-A, which collapse growth cones via the RhoA/ROCK signaling pathway (Weidner et al., 2025; Hassanli et al., 2023); meanwhile, adult CNS neurons have low intrinsic regenerative capacity, with insufficient induction of regeneration-associated genes and enhanced activity of growth-inhibitory signaling pathways such as PTEN/mTOR (Conklin et al., 2026; Guo et al., 2019). Oligodendrocyte death causes extensive axonal demyelination, differentiation of residual oligodendrocyte progenitor cells into mature oligodendrocytes is impeded, and insufficient neo-myelination decreases axonal conduction velocity and predisposes axons to degeneration (Chen et al., 2023; Kong et al., 2025). If myelin debris is not cleared promptly, it persistently activates immune cells and releases inhibitory molecules, forming a vicious cycle of debris accumulation, chronic inflammation, and blocked regeneration (Kong et al., 2025; Liu Z. et al., 2025). In addition, impaired autophagic flux after injury prevents the effective clearance of damaged mitochondria and misfolded proteins, and intracellular metabolic waste accumulation further exacerbates the functional exhaustion of neurons and glial cells, whereas moderately activated autophagy is protective in maintaining cellular homeostasis and promoting repair; dysregulation of this process constitutes an important intrinsic factor in the prolongation of secondary injury into the chronic phase (Li RY. et al., 2023; Zhao et al., 2026) (Figure 1).

**FIGURE 1:**
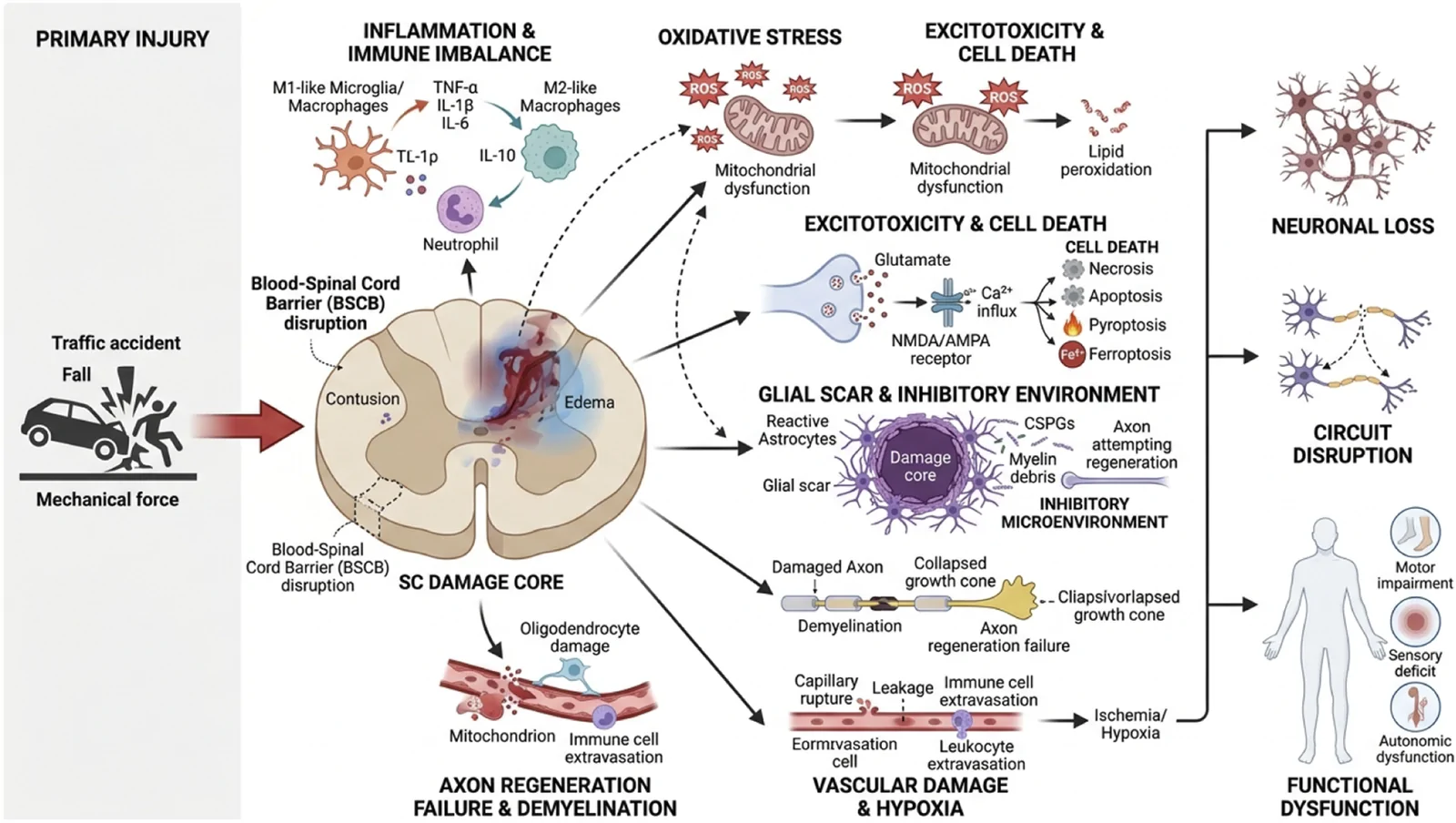
Pathophysiological mechanisms of spinal cord injury. Following SCI, the primary mechanical insult directly causes contusion, compression, or transection of spinal cord tissue, accompanied by hemorrhage, edema, cell destruction, and disruption of the blood-spinal cord barrier. On this basis, secondary injury cascades are rapidly initiated and progressively aggravate local microenvironmental imbalance, mainly including: (1) inflammatory response and immune microenvironment dyshomeostasis, manifested by microglial activation, infiltration of peripheral neutrophils and monocytes/macrophages, enhanced M1 pro-inflammatory polarization, and inflammasome-mediated pyroptotic amplification; (2) enhanced oxidative stress, in which mitochondrial dysfunction leads to massive ROS generation, further inducing lipid peroxidation, DNA damage, and membrane destruction; (3) excitotoxicity and activation of multiple cell death modalities, including excessive glutamate release, overactivation of NMDA/AMPA receptors, Ca2+ overload, and apoptosis, necrosis, pyroptosis, and ferroptosis; (4) vascular damage and persistent blood-spinal cord barrier disruption, resulting in plasma exudation, immune cell infiltration, local ischemia-hypoxia, and edema; (5) reactive astrogliosis and extracellular matrix remodeling forming the glial scar, accompanied by deposition of CSPGs and myelin debris, constituting an inhibitory microenvironment for axonal regeneration; and (6) oligodendrocyte damage and demyelination leading to impaired axonal conduction and further aggravation of regenerative failure. These pathological processes interact with and amplify one another, ultimately resulting in neuronal loss, neural circuit interruption, blocked axonal regeneration, and motor, sensory, and autonomic dysfunction.

## 3 Types and characteristics of nanocarrier-based drug delivery systems

Given the complex pathological microenvironment of SCI, researchers have developed various types of nanocarrier-based drug delivery systems. Different carriers have respective strengths in structural composition, drug-loading modalities, *in vivo* behavior, and functional properties, providing diversified options matched to different therapeutic scenarios, such as anti-inflammatory and antioxidative treatment in the acute phase, neuroprotection in the subacute phase, and regenerative repair in the chronic phase (Khan et al., 2020; Li et al., 2025; Lu et al., 2023). A stage-by-stage mapping of these platforms onto the pathological evolution of SCI, together with an assessment of the strength of the supporting evidence (Table 1).

**TABLE 1:** Stage-specific positioning of nanocarrier platforms across the pathological phases of spinal cord injury.

Pathological stage	Dominant pathology	Most suitable platforms	Representative strategies	Typical administration routes	Evidence strength
Acute (0–72 h)	Neuroinflammation, oxidative burst, BSCB disruption, excitotoxicity	Liposomes; polymeric nanoparticles; antioxidative nanozymes	Biomimetic minocycline-dextran sulfate liposomes; CAQK-modified rosuvastatin liposomes; polyphenol-metformin nanoparticles; cerium oxide nanozymes	Mainly intravenous; intrathecal	Moderate: multiple independent rat studies, but nearly all with immediate post-injury dosing
Subacute (3 d–2 wk)	Apoptosis, glial activation, demyelination, persistent inflammation	Exosomes and biomimetic carriers; immunomodulatory hydrogels; microneedle patches	Angiopep-2-engineered exosomes; miR-124–3p mesoporous silica; IL-4@ZIF-8 hydrogel; nanodrug-loaded microneedles	Local/intrathecal; intranasal; intravenous	Moderate but heterogeneous: few delayed-treatment designs; potency assays vary across studies
Chronic (>2 wk)	Glial scarring, cystic cavitation, axonal regeneration failure	Nanohydrogels and composite scaffolds; aligned fiber scaffolds; pre-vascularized tissue-engineered constructs	Biomimetic spinal cord fiber scaffolds; 3D-printed multichannel scaffolds; individually tailored modular hydrogels	Surgical implantation; minimally invasive injection	Low to moderate: mostly severe rodent injuries; outcomes rarely reported beyond 8–12 weeks

### 3.1 Liposomes

Liposomes are spherical vesicles composed of a phospholipid bilayer enclosing an aqueous core. With good biocompatibility and biodegradability, they possess both a hydrophilic core and a lipophilic bilayer and can encapsulate water-soluble and lipid-soluble drugs simultaneously (An et al., 2022; Lu et al., 2023). In a rat SCI model, macrophage membrane-biomimetic nanoliposomes co-loading minocycline and dextran sulfate targeted inflammatory cells in the lesion, significantly downregulated TNF-alpha and IL-1beta expression and reduced neuronal apoptosis by inhibiting Toll-like receptor signaling and the nuclear factor-kappaB (NF-kappaB) pathway, demonstrating the prominent advantage of biomimetic liposomes in inflammation-targeted therapy (An et al., 2022). CAQK peptide-modified rosuvastatin liposomes bound proteoglycans in the injured region and preferentially accumulated in foam-like macrophages phagocytosing myelin debris in a rat SCI model, effectively interrupting the vicious cycle of debris phagocytosis and inflammatory amplification and significantly mitigating secondary injury (Liu Z. et al., 2025). A drug-loaded liposome-hydrogel combination system achieved spatiotemporally sequential immunomodulation and neurogenesis in SCI rats, indicating that combining liposomes with scaffold materials can synergistically enhance therapeutic efficacy (Zhang W. et al., 2026). Furthermore, nanoformulated curcumin liposomes, by either local or systemic administration, modulated the local inflammatory response, attenuated glial activation, and promoted motor recovery in a rat SCI model, indicating that liposomes are suitable for the solubilization and delivery of active components of traditional Chinese medicine (Krupa et al., 2019; Ahmadpour et al., 2025). In clinically related research, nanocurcumin showed favorable anti-inflammatory effects compared with dexamethasone and coenzyme Q10 against the inflammatory response after lumbar laminectomy, suggesting the translational potential of nanoformulated Chinese herbal active constituents as adjunctive therapy in spinal cord-related surgery (Mousavi et al., 2022). A targeted nanosystem based on mesoporous polydopamine delivering 8-gingerol significantly alleviated secondary injury by inhibiting the inflammasome pathway in a rat SCI model, indicating that combining mesoporous carriers with natural polyphenolic active molecules can achieve synergistic anti-inflammation (Liu G. et al., 2024). The main limitation of liposomes is their susceptibility to clearance by the mononuclear phagocyte system during circulation, which often requires PEGylation or cell membrane coating strategies for improvement (An et al., 2022; Zhang W. et al., 2026).

### 3.2 Polymeric nanoparticles

Polymeric nanoparticles are prepared from natural or synthetic polymeric materials, including poly(lactic-co-glycolic acid) (PLGA), polyethylene glycol (PEG), chitosan, hyaluronic acid, and silk fibroin (Waris et al., 2022; Nejati-Koshki et al., 2017). Their core advantage lies in excellent sustained-release performance: drug release kinetics can be precisely controlled through the material degradation rate, maintaining therapeutic drug concentrations at the lesion for up to several weeks, and their surfaces are amenable to conjugation with targeting ligands or responsive groups (Bin et al., 2017; Wan et al., 2024). In a rat spinal cord hemisection model, albumin-conjugated chitosan nanoparticles co-delivering methylprednisolone and minocycline markedly enhanced targeted drug accumulation in the lesion, suppressed inflammatory cytokine expression, and reduced neuronal loss more effectively than the free drugs, demonstrating that targeted delivery by polymeric nanoparticles can significantly improve the efficacy of conventional anti-inflammatory drugs (Bin et al., 2017). In a model of SCI-associated neuropathic pain, PEG-PLGA nanoparticles delivered the analgesic peptide CBD3A6K-RhB intrathecally to achieve long-lasting pain relief, indicating the applicability of polymeric nanoparticles to the treatment of SCI complications (Zhang Z. et al., 2026). Self-assembled nanoparticles of natural polyphenols and metformin exerted dual anti-inflammatory and antioxidative effects through AMPK pathway activation in a rat SCI model and significantly improved motor function, showing that multi-component self-assembly enables multi-pathway synergistic intervention (Yuan et al., 2023). In addition, carrier-free nanoparticles co-assembled from a methylprednisolone dimer and rutin avoided carrier material-related safety concerns while achieving a synergistic anti-inflammatory and antioxidative effect in SCI rats (Wang et al., 2023).

### 3.3 Polymeric micelles

Polymeric micelles are formed by the self-assembly of amphiphilic block copolymers or amphiphilic molecules in water into core-shell structures; the hydrophobic core solubilizes hydrophobic drugs, while the hydrophilic shell maintains dispersion stability (Wang et al., 2019; Kartha et al., 2020). In a rat SCI model, polysialic acid-based micelles exploited the natural affinity between polysialic acid and neural cell adhesion molecules to achieve regeneration-oriented targeted delivery, significantly promoting axonal extension and improving motor function, indicating that micellar carriers can achieve precise targeting through bio-specific ligands (Wang et al., 2019). Phospholipid micelles encapsulating a phospholipase A2 inhibitor abolished pain behavior in a neuropathic pain model, demonstrating the practical value of micelles in treating SCI complications (Kartha et al., 2020). Paclitaxel-idebenone prodrug self-assembled nanoparticles combined pro-axonal-regeneration and antioxidative functions in SCI rats and significantly promoted functional recovery, indicating that a heterodimeric prodrug strategy can endow micelles with multifunctionality (Xu et al., 2024). Curcumin-polypeptide conjugate nanoassemblies targeted and scavenged reactive aldehydes derived from lipid peroxidation in an SCI model, interrupting the secondary amplification of oxidative damage, demonstrating that functionalized micelles can precisely eliminate specific pathological molecules (Liu Y. et al., 2024).

### 3.4 Inorganic nanozymes

Inorganic nanozymes are functional carriers that mimic the catalytic activities of natural enzymes. Owing to their resistance to enzymatic degradation in biological environments, their repeated catalytic capability, and their ease of functional modification, they occupy a unique position in oxidative stress intervention after SCI. Cerium oxide nanoparticles possess both superoxide dismutase- and catalase-like activities; when loaded on chitosan in a rat SCI model, they displayed significant neuroprotective and pro-regenerative effects with enhanced autocatalytic regeneration, indicating that cerium-based nanozymes can achieve long-lasting neuroprotection through sustained ROS scavenging (Fang and Song, 2019; Dong et al., 2020). Cerium oxide nanoparticles combined with photobiomodulation synergistically alleviated pain and promoted functional recovery in SCI rats, indicating that combining nanozymes with physical therapy can produce synergistic effects (Behroozi et al., 2024). Prussian blue nanozymes scavenged free radicals through multi-enzyme cascades and coordinately regulated zinc homeostasis in SCI models, significantly attenuating ferroptosis-related lipid peroxidation, indicating that Prussian blue analogues can simultaneously intervene in oxidative stress and metal ion imbalance (Gao J. et al., 2024; Wang et al., 2026). A zinc-based metal-organic framework (MOF) aggregation-induced emission nanozyme integrated catalytic activity with real-time imaging in SCI rats, providing a new platform for theranostics (Zheng et al., 2024). The main limitation of inorganic nanozymes lies in the risk of long-term accumulation of metal ions such as cerium and zinc in organs including the brain and liver; current toxicological data are mostly limited to subacute observations, and the chronic accumulation safety threshold beyond 90 days has not been established (Singh et al., 2026; Fang and Song, 2019).

### 3.5 Other inorganic nanomaterials

Beyond nanozymes, mesoporous materials and two-dimensional nanomaterials, with their high specific surface area and tunable pore structures, provide another functional platform for SCI therapy. Mesoporous silica nanoparticles loaded with miRNA-124–3p and disguised with stem cell membranes achieved lesion-targeted gene delivery in an SCI model, driving microglia toward a pro-repair phenotype, indicating that mesoporous carriers combined with biomimetic camouflage can markedly enhance the efficacy of gene drugs (Fan X. et al., 2024). Graphene oxide-immobilized chondroitinase ABC efficiently degraded inhibitory CSPGs in an SCI model, eliminating the chemical inhibitory barrier of the scar, indicating that the large surface area of two-dimensional materials favors enzyme stabilization and delivery (Hassanli et al., 2023). MXene films mediated wireless magnetoelectric stimulation to promote neural stem cell differentiation in an SCI model, indicating that conductive two-dimensional materials can couple physical stimulation with drug delivery (Hu et al., 2025). Graphene-based composites combined with local delivery of cerebrolysin promoted neural repair in SCI models, showing that the large surface area of graphene is suitable for multi-component synergistic delivery (Yari-Ilkhchi et al., 2024; Girão et al., 2022). The functional versatility of mesoporous silica and graphene-based materials offers a unique platform for theranostics and physicochemical synergistic therapy (Niu et al., 2026; Zha et al., 2025); however, their long-term *in vivo* accumulation and metabolic safety remain questions that must be answered before translation, particularly as the degradation behavior and long-term neurotoxicity of graphene-based materials in neural tissue are still debated (Girão et al., 2022).

### 3.6 Exosomes and biomimetic nanocarriers

Exosomes are extracellular vesicles approximately 30–150 nm in diameter secreted by cells; they naturally carry active molecules of parental cells, including proteins, mRNAs, and miRNAs, and offer low immunogenicity, natural barrier-crossing ability, and inherent tropism (Su et al., 2024; Mou et al., 2025). In a rat SCI model, exosomes from hypoxia-preconditioned mesenchymal stem cells (MSCs) exerted anti-inflammatory, pro-angiogenic, and neuroprotective effects by carrying miR-216a-5p and significantly improved motor function, indicating that hypoxic preconditioning can enhance the therapeutic potential of exosomes (Liu W. et al., 2020). Angiopep-2 peptide-modified M2 microglial exosomes targeted the LRP-1-positive injured region in SCI rats and promoted remyelination and maintenance of BSCB integrity, indicating that engineering modification can endow exosomes with precise targeting ability (Kong et al., 2025). Melatonin-primed MSC exosomes inhibited neuronal apoptosis and improved motor function by delivering anti-apoptotic proteins such as USP29 in an SCI model, indicating that drug preconditioning can markedly enhance the neuroprotective potency of exosomes (Liu W. et al., 2021). miR-672–5p carried by anti-inflammatory microglial exosomes indirectly promoted myelin preservation by suppressing inflammatory pathways in SCI rats, indicating that functional miRNAs carried by exosomes are key effector molecules of repair (Zhou et al., 2022). A Schwann cell-derived exosome and methylprednisolone composite patch integrated exosome therapy with a classic anti-inflammatory drug at the level of local delivery in an SCI model, exerting synergistic repair effects and indicating the clinical translation prospects of combining exosomes with conventional drugs (Zhu et al., 2023). A three-dimensional (3D)-cultured MSC exosome-hydrogel microneedle patch achieved minimally invasive local delivery of exosomes in an SCI model, indicating that 3D culture can enhance the therapeutic potency of exosomes (Han et al., 2022). Induced pluripotent stem cell (iPSC)-derived exosomes prevented neural stem cell senescence and promoted repair in an SCI model, indicating the unique advantage of iPSC-derived exosomes in maintaining stem cell vitality (Chen et al., 2025). Antler blastema progenitor cell-derived exosomes displayed pro-repair activity in an SCI model, indicating that the lineage sources of exosomes can be extended to tissues endowed with natural regenerative capacity (Yang et al., 2025). Regarding biomimetic nanocarriers, macrophage membrane-coated nanoliposomes and microglial membrane-coated zinc sulfide nanoparticles achieved lesion-homologous targeting and immune evasion in SCI models, indicating that cell membrane coating strategies can endow synthetic nanoparticles with the homing ability of natural biological membranes (An et al., 2022; Qin et al., 2025). Neutrophil membrane modification strategies likewise showed good lesion tropism in inflammation-targeted delivery (An et al., 2022; Kim et al., 2018). Engineered biomimetic nanosponges acted as cytokine traps while simultaneously scavenging ROS in an SCI model, indicating that biomimetic carriers can neutralize multiple pro-inflammatory signals at once (Yang et al., 2024). The main challenges for such carriers are yield, purification uniformity, and the establishment of quality control standards (Lai et al., 2022; Li Q. et al., 2026).

### 3.7 Nanohydrogels and composite scaffolds

Nanohydrogels and composite scaffolds fill lesion cavities in the form of injectable gels or implantable scaffolds, serving as local depots for the long-term sustained release of nanoformulations, cells, and factors, while providing mechanical support and tissue bridging (Santi et al., 2021; Ju et al., 2022). In a rat SCI model, a manganese dioxide nanoparticle-dotted hydrogel catalytically decomposed excessive ROS *in situ* and released oxygen, significantly improving the survival microenvironment for stem cell transplantation, indicating that hydrogel-loaded nanozymes can simultaneously address oxidative stress and ischemia-hypoxia (Li et al., 2019). A hyaluronic acid hydrogel containing IL-4-loaded zeolitic imidazolate framework-8 (ZIF-8) nanoparticles continuously released IL-4 to drive macrophage M2 polarization and synergize with neural induction in an SCI model, indicating that immunomodulatory hydrogels can couple microenvironment remodeling with neural regeneration (Xin et al., 2022). An adaptive phenol-based hydrogel combined with metformin nanoparticles targeted the core pathways of oxidative stress and ferroptosis in SCI rats and significantly promoted spinal cord repair, indicating that responsive hydrogels can precisely intervene in specific modalities of cell death (Feng et al., 2025). A nitroquinoline nanodrug-loaded microneedle patch re-established microglial homeostasis and achieved scar-reduced repair in an SCI model, indicating that microneedle delivery enables minimally invasive, long-lasting local drug administration (He et al., 2025). With respect to composite scaffolds, biomimetic nanofiber scaffolds guided directed axonal regeneration and achieved functional reconstruction across the lesion in SCI rats, indicating that physically guided structures can bypass the inhibitory microenvironment and provide directional growth channels for axons (Chen et al., 2023; Usmani et al., 2020). Multi-channel scaffolds constructed by 3D printing achieved precise factor delivery and pre-vascularized spinal cord-like tissue engineering constructs in SCI models, indicating that 3D printing enables customization to match complex lesion morphology (Cao et al., 2025; Khaledian et al., 2024; Fan C. et al., 2024). A neonatal spinal cord-like scaffold with hierarchical structure and neurogenetic microenvironment supported the repair of long-segment defects in a rat SCI model, indicating that the microstructural design of biomimetic scaffolds can mimic developmental neural niches (Bai et al., 2026). Biodegradable conductive melanin-poly(3-hydroxybutyrate) fibrous scaffolds provided electrical signal support while avoiding permanent implant residues in an SCI model, indicating that biodegradable conductive materials can balance electrophysiological modulation with biosafety (Agrawal et al., 2022). Acellular spinal cord matrix scaffolds combined with sustained VEGF release promoted vascularized remodeling in SCI rats, indicating that combining a natural matrix template with growth factor sustained release can synchronize structural and vascular repair (Xu Z. X. et al., 2017). Magnesium-aluminum layered double hydroxide nanoparticles reprogrammed the immune microenvironment and promoted neurogenesis by intervening in TGF-beta signaling in a complete transection SCI model, indicating that inorganic nanomaterials themselves can act as immunomodulators (Zhu et al., 2021). A zinc-manganese-doped silk fibroin hydrogel amplified the expression of metalloregulatory proteins in macrophages and promoted the anti-inflammatory repair phenotype in an SCI model, indicating that the intrinsic ion-releasing properties of materials can serve as an internal driving force for immunomodulation (Han et al., 2024) (Table 2; Figure 2).

**TABLE 2:** Summary of the types and characteristics of nanocarrier-based drug delivery systems.

Carrier type	Representative materials	Main features	Loading/application examples	Main limitations
Liposomes	Phospholipid bilayer vesicles	Good biocompatibility; capable of encapsulating both water-soluble and lipid-soluble drugs	Biomimetic minocycline-dextran sulfate liposomes; CAQK-modified rosuvastatin liposomes	Readily cleared by the mononuclear phagocyte system *in vivo*; circulation time prolonged by PEGylation but still <24 h; poor storage stability with a tendency toward drug leakage
Polymeric nanoparticles	PLGA, chitosan, PEG	Excellent sustained-release performance; highly modifiable surface	Methylprednisolone-minocycline chitosan nanoparticles; polyphenol-metformin nanoparticles	Drug loading capacity usually <10%; acidic microenvironment generated by PLGA degradation products may aggravate local inflammation; burst release affects release uniformity
Polymeric micelles	Amphiphilic block copolymers	Hydrophobic core solubilizes poorly soluble drugs; good dispersion stability	Polysialic acid-based micelles; paclitaxel-idebenone prodrug micelles	Prone to disassembly when diluted in blood below the critical micelle concentration; *in vivo* stability usually <6 h; drug loading limited by the molecular weight of the hydrophobic block
Inorganic nanozymes	Cerium oxide, Prussian blue, zinc-based MOFs	Persistent catalytic activity with repeated ROS scavenging; imaging capability	Cerium oxide nanozymes; Prussian blue nanozymes; zinc-based AIE nanozymes	Accumulation of metal ions such as cerium and zinc in the brain and liver; the chronic accumulation safety threshold beyond 90 days has not been established; catalytic activity affected by microenvironmental pH and temperature
Other inorganic nanomaterials	Mesoporous silica, graphene oxide, MXene	High specific surface area; tunable pore structure; electrical conductivity and magneto-optical responsiveness	Mesoporous silica-miR-124 delivery; graphene oxide-chondroitinase ABC; MXene films	Degradation behavior of graphene-based materials in neural tissue unclear and long-term neurotoxicity controversial; *in vivo* degradation of mesoporous silica usually takes months
Exosomes and biomimetic carriers	MSC-derived exosomes; cell membrane-coated nanoparticles	Low immunogenicity; natural barrier-crossing ability; inherent tropism	Angiopep-2-engineered exosomes; macrophage membrane-coated liposomes	Low yield, typically only microgram quantities of exosomes per liter of culture medium; poor batch-to-batch consistency of membrane protein orientation; lack of unified quality control standards
Nanohydrogels and composite scaffolds	Hyaluronic acid, silk fibroin, collagen hydrogels	Fill lesion cavities; mechanical support; long-term sustained release	MnO2 hydrogel; IL-4@ZIF-8 hydrogel; microneedle patches	Require surgical implantation or minimally invasive puncture with a risk of infection; difficult matching of degradation rate to the tissue repair timeline-overly rapid degradation causes secondary cavitation, whereas overly slow degradation hinders tissue remodeling

**FIGURE 2:**
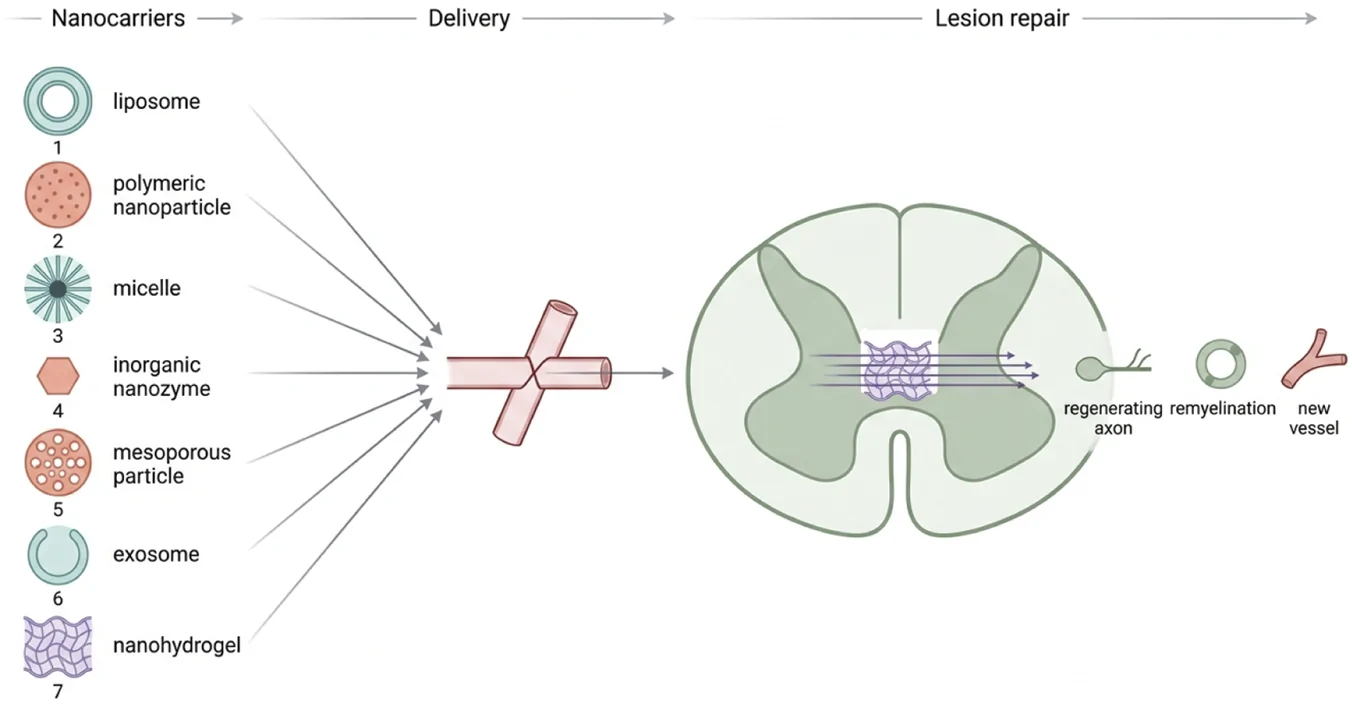
Schematic overview of nanocarrier-based drug delivery for spinal cord injury. Seven major carrier classes (1, liposomes; 3, polymeric micelles; 6, exosomes—vesicle-type; 2, polymeric nanoparticles; 4, inorganic nanozymes; 5, mesoporous and 2D materials—particle-type; 7, nanohydrogels and composite scaffolds—matrix-type) converge through circulation and the blood-spinal cord barrier to the lesion cavity, where hydrogel depots and aligned scaffolds bridge the gap and support axonal regeneration, remyelination, and revascularization.

## 4 Mechanisms of nanocarrier-based drugs in the treatment of spinal cord injury

Mechanisms are discussed below in the order of the therapeutic target addressed, namely, inflammation and immune polarization, oxidative stress, apoptosis and programmed cell death, axonal regeneration, myelin repair, glial-scar remodelling, and vascular repair, and for each target the principal evidence linking it to functional recovery is traced. Carrier-specific materials science, including fabrication, ligand chemistry and pharmacokinetics, is covered only where it directly determines a mechanism. The closing discussion places these mechanisms on the temporal axis of SCI and appraises the strength of the supporting evidence.

### 4.1 Suppression of inflammation and regulation of immune polarization

Suppression of neuroinflammation is the most direct and most extensively studied mechanism of nanocarrier-based therapy for SCI, while M1/M2 polarization imbalance of macrophages and microglia is the hub linking chronic inflammation and stalled repair; the two constitute two levels of the same immunoregulatory axis. Nanocarriers increase local concentrations of anti-inflammatory drugs through targeted delivery. Building on the carrier characteristics detailed, targeted nanocarriers markedly increase local anti-inflammatory drug concentrations. Albumin-conjugated chitosan nanoparticles co-loading methylprednisolone and minocycline, biomimetic liposomes co-delivering minocycline and dextran sulfate, and lesion-homing rosuvastatin liposomes all significantly suppressed the expression of TNF-alpha, IL-1β, and IL-6 in rat SCI models (Bin et al., 2017; An et al., 2022; Liu Z. et al., 2025); mechanistically, these effects converge on inhibition of Toll-like receptor/NF-kappaB signaling and on precise targeting of pathological cell subsets such as foam-like macrophages, rather than on non-specific drug accumulation.

Regarding polarization regulation, in addition to directly delivering anti-inflammatory drugs, nanocarriers drive the shift of macrophages and microglia from the M1 pro-inflammatory phenotype to the M2 repair phenotype by delivering polarization modulators and by tuning the physicochemical properties of the materials themselves.The IL-4@ZIF-8 hydrogel described above couples M2 polarization with neural induction (Xin et al., 2022), indicating that local delivery of polarization modulators can effectively remodel the immune microenvironment. Metformin nanoparticles and polyphenol self-assembled nanoparticles promoted a metabolic anti-inflammatory phenotypic switch through AMPK pathway activation in SCI models, indicating that nanoformulation of small-molecule drugs can enhance their immunoregulatory potency (Feng et al., 2025; Yuan et al., 2023). The layered double hydroxide nanoparticles and the zinc-manganese-doped silk fibroin hydrogel described above act on the immune microenvironment through TGF-beta signaling and ion release, respectively (Zhu et al., 2021; Han et al., 2024), indicating that the physicochemical properties of the materials themselves can be an internal driving force for immunomodulation. Carbon dot-integrated hydrogels modulated inflammation by regulating macrophage metabolic reprogramming in an SCI model, indicating that metal-free carbon-based materials also possess immunoregulatory capacity (Hu et al., 2026).

Regarding exosome-mediated indirect polarization, although direct immunomodulation by synthetic nanoparticles and inorganic materials is highly effective, exosomes, by virtue of their naturally carried miRNA and protein cargo, can reprogram immune cell phenotypes in a manner closer to physiological regulation. In a rat SCI model, exosomes from hypoxia-preconditioned MSCs carried miR-216a-5p to reprogram pro-inflammatory microglia toward an anti-inflammatory phenotype and significantly improved motor function, indicating that functional miRNAs are the key molecules controlling the direction of polarization (Liu W. et al., 2020). The anti-inflammatory microglial exosomes carrying miR-672–5p described above likewise suppress inflammatory pathways, indicating that exosomal miRNA cargo can simultaneously regulate inflammation and tissue repair (Zhou et al., 2022). The Angiopep-2-engineered microglial exosomes and melatonin-primed MSC exosomes described above also downregulate inflammatory pathways through miRNA-mediated mechanisms, exerting both central and peripheral anti-inflammatory effects (Kong et al., 2025; Liu W. et al., 2021). Exosomes from hypoxia-preconditioned M2 macrophages improved repair through both pro-angiogenic and immunomodulatory routes in SCI models (Huang et al., 2022; Li et al., 2022). Regarding downstream effector clearance, a nanozyme-switched efferocytosis initiation platform broke the persistent inflammatory cycle by initiating macrophage clearance of apoptotic cells and reprogrammed the pathological network in an SCI model, indicating that promoting efferocytosis is an effective strategy for interrupting the chronicization of inflammation (Li C. et al., 2026). The nitroquinoline-loaded microneedle patch described above likewise restores microglial homeostasis and reduces scar formation in a minimally invasive manner (He et al., 2025). Multidimensionally engineered exosomes achieved systematic design of targeted delivery and microglial reprogramming in an SCI model, indicating that engineering modification can simultaneously optimize the targeting and immunoregulatory functions of exosomes (Xiong W. et al., 2025). Nanoformulated fingolimod downregulated sphingosine-1-phosphate receptor signaling in a rat SCI model, significantly inhibiting NLRP3 inflammasome assembly, caspase-1 activation, and IL-1beta maturation, and reducing glial activation and cavity expansion, indicating that targeting the inflammasome assembly step can suppress the inflammatory cascade at its source (M Alsharbaty et al., 2026). Engineered biomimetic nanosponges acted as cytokine traps while scavenging ROS in an SCI model, indicating that biomimetic carriers can simultaneously neutralize multiple pro-inflammatory signals (Yang et al., 2024). Collectively, these studies demonstrate that nanocarriers, through targeted accumulation, responsive release, and multi-pathway blockade, possess significant advantages over free drugs in the spatiotemporal regulation of neuroinflammation and immune polarization (Figure 3).

**FIGURE 3:**
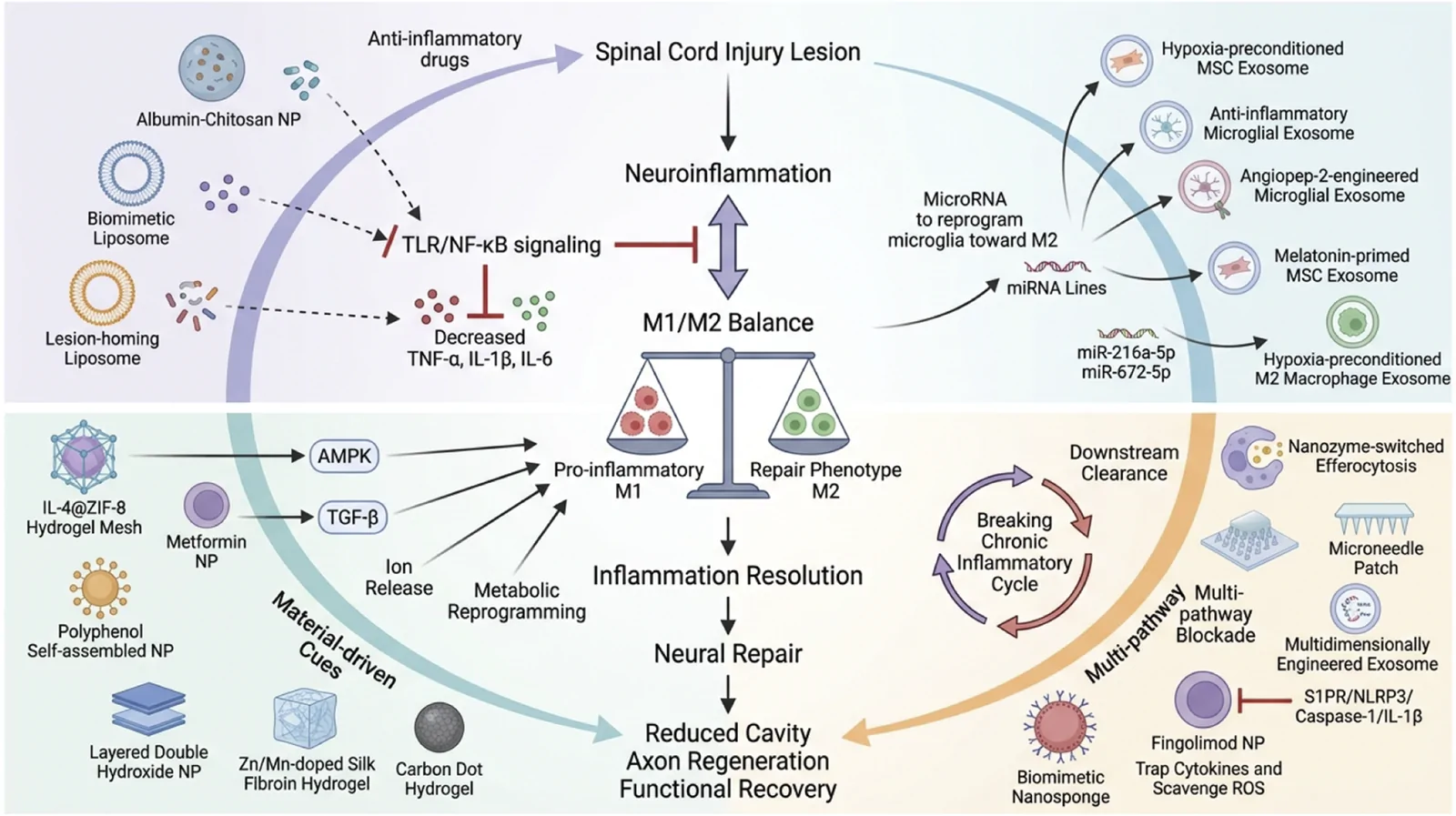
Nanocarriers suppress neuroinflammation and rebalance M1/M2 polarization through a shared immunoregulatory axis in SCI. Following SCI, neuroinflammation suppression and M1/M2 polarization constitute two levels of the same immunoregulatory axis. Left: targeted nanocarriers deliver anti-inflammatory drugs, inhibit TLR/NF-κB signaling, and reduce TNF-α, IL-1β, and IL-6; polarization modulators and intrinsic material cues drive M1→M2 via AMPK, TGF-β, ion release, and metabolic reprogramming. Right: engineered exosomes reprogram microglia via miRNAs; downstream efferocytosis, NLRP3/caspase-1/IL-1β blockade, cytokine trapping, and ROS scavenging break the chronic inflammatory cycle. All pathways converge on the central M1/M2 balance to promote inflammation resolution, cavity reduction, axon regeneration, and functional recovery.

### 4.2 Attenuation of oxidative stress

In response to the storm of reactive oxygen and nitrogen species after SCI, nanocarrier-based delivery systems have evolved three major strategies: enzyme replacement, catalytic scavenging, and responsive drug release. Regarding the nanoformulation of antioxidant enzymes, co-loading superoxide dismutase and catalase in nanocarriers protected enzyme activity, prolonged the half-life, and achieved lesion accumulation in a rat SCI model, significantly attenuating mitochondrial oxidative damage and neuronal apoptosis, indicating that nanoformulation effectively overcomes the short half-life and poor stability of natural enzymes (Andrabi et al., 2020). Regarding nanozyme-mediated catalytic scavenging, the cerium oxide and Prussian blue platforms introduced above persistently scavenge ROS through the Ce3+/Ce4+ redox cycle and multi-enzyme cascade catalysis, respectively, while coordinately regulating zinc homeostasis (Fang and Song, 2019; Dong et al., 2020; Gao J. et al., 2024; Wang et al., 2026). A nitric oxide-releasing hollow cerium oxide nanozyme-hydrogel composite system scavenged ROS while releasing nitric oxide to improve blood flow and synergized with neural stem cell transplantation to promote repair in an SCI model, indicating that combining nanozymes with gaseous signaling molecules enables simultaneous intervention in oxidative stress and ischemia-hypoxia (Liu D. et al., 2025). Diselenide nanogels and a zinc-based MOF aggregation-induced emission nanozyme integrated catalytic activity with real-time imaging monitoring in SCI rats, providing a new platform for theranostics (Zheng et al., 2024; Ma et al., 2026). Selenized neural stem cell exosomes exerted protective effects by enhancing the antioxidant enzyme system in an SCI model, indicating that chemical modification can confer additional antioxidative capacity on biological carriers (Wang et al., 2025). Regarding microenvironment-responsive antioxidative delivery, ROS-responsive phenylborate ester nanodrugs released drugs specifically in regions of high oxidative stress (Liu Y. et al., 2020). ROS-responsive mesoporous silica nanoparticles delivered a DUSP26 inhibitor in a rat SCI model, scavenging ROS while relieving the inhibition of ERK regenerative signaling, indicating that responsive delivery can achieve dual antioxidant and pro-regenerative effects (Zhang et al., 2025). ROS-responsive trifluoperazine prodrug nanoparticles and MSC exosomes synergistically delivered precise intervention to the lesion core in an SCI model, indicating that combining prodrug nanoparticles with stem cell therapy can cover a broader range of pathological processes (Cheng et al., 2026). Aldehyde-scavenging polypeptide-curcumin conjugate nanoassemblies targeted reactive aldehydes derived from lipid peroxidation in an SCI model and cut off the secondary amplification of oxidative damage, indicating that targeting downstream toxic products is an important supplement to antioxidant strategies (Liu Y. et al., 2024). A neuron-targeted ROS regulation system provided new ideas for emergency treatment in an acute SCI model, indicating that neuron-specific targeting can improve the precision of antioxidant therapy in the acute phase (Ying et al., 2025). Systems such as nanozyme-catalyzed hydrogen production and a hydrogen sulfide donor zinc-citrate MOF integrated gasotransmitter therapy with antioxidation and suppressed downstream inflammatory and cell death pathways in SCI models, indicating that the controlled release of gaseous signaling molecules adds a new dimension to oxidative stress intervention (Ding et al., 2026; Yao et al., 2025; Gao X. et al., 2024).

### 4.3 Inhibition of neuronal apoptosis

Nanocarriers achieve neuroprotection through multi-pathway coordinated inhibition of secondary neuronal apoptosis. Anti-inflammatory and antioxidative nanotherapies indirectly reduce apoptotic triggers by lowering upstream injury signals; antioxidant enzyme nanoparticles significantly reduced caspase-3 activation and neuronal apoptosis by attenuating mitochondrial oxidative stress in a rat SCI model (Andrabi et al., 2020). Regarding the direct delivery of anti-apoptotic and neurotrophic molecules, a collagen-binding HGF sustained-release system inhibited neuronal apoptosis and promoted synaptic plasticity by activating the PI3K/Akt survival signaling pathway in a rat SCI model, indicating that targeted sustained release of growth factors can effectively activate endogenous neuroprotective programs (Yamane et al., 2019; Yamane et al., 2018). A nanoformulated BDNF delivery system inhibited neuronal apoptosis and improved motor function through survival pathway activation in an SCI model, indicating that the nanoformulation of neurotrophic factors can overcome their short half-life and poor barrier-crossing capacity (Xia et al., 2021). An ROS-responsive paclitaxel prodrug nanomedicine stabilized microtubules at a low dose, inhibited apoptosis, and promoted axonal extension in a rat SCI model, indicating that the prodrug strategy can achieve both neuroprotection and pro-regeneration while reducing systemic toxicity (Xu et al., 2024). Regarding paracrine protection by stem cell exosomes, melatonin-primed MSC exosomes inhibited neuronal apoptosis in the lesion and improved motor function by delivering anti-apoptotic proteins such as USP29 in SCI rats, indicating that exosomes can serve as natural delivery vehicles for anti-apoptotic molecules (Liu W. et al., 2021; Zhai et al., 2021). Adipose-derived stem cell exosomes and placental MSC exosomes combined with hyperbaric oxygen both significantly reduced apoptotic indices in SCI models, indicating that combining exosomes with physical therapies can produce synergistic anti-apoptotic effects (Harrell et al., 2022; Cheshmi et al., 2023). Hypoxic preconditioning enhanced the loading of pro-survival miRNAs in exosomes and improved their anti-apoptotic potency, indicating that preconditioning strategies can directionally optimize exosome cargo composition (Liu W. et al., 2020; Li et al., 2022). Baicalein-functionalized collagen scaffolds indirectly reduced cell loss by modulating the neuronal differentiation microenvironment in an SCI model, indicating that scaffold materials can provide both structural support and anti-apoptotic signals (Qian et al., 2023). An MSC lysate sustained-release nanohydrogel attenuated tissue destruction by inhibiting inflammatory cell death in SCI rats, indicating that whole-cell lysates retain multiple protective components (Sun M. L. et al., 2026).

### 4.4 Regulation of programmed cell death

The discovery and nanoscale regulation of regulated cell death modalities other than apoptosis represent the current frontier of this field. Regarding ferroptosis, iron overload and glutathione peroxidase 4 inactivation after SCI drive lipid peroxidation-dependent cell death. An iron-chelating and hydrogen-releasing microneedle patch simultaneously scavenged free iron and ROS, dually inhibiting ferroptosis and significantly promoting spinal cord repair in a rat SCI model, indicating that targeting the key drivers of ferroptosis can achieve synergistic therapeutic effects (Ding et al., 2026). A metformin nanoparticle-hydrogel system targeted the core pathways of oxidative stress and ferroptosis in an SCI model, indicating that combining nanocarrier-based therapeutics with hydrogels enables sustained intervention in ferroptosis (Feng et al., 2025). Regarding pyroptosis, the NLRP3 inflammasome-caspase-1-GSDMD pathway mediates pyroptotic amplification of the inflammatory cascade; nanoformulated fingolimod significantly inhibited this pathway and reduced pyroptosis-related release in a rat SCI model, indicating that blocking pyroptosis at the inflammasome level can effectively curb inflammatory amplification (M Alsharbaty et al., 2026). Regarding autophagy and mitophagy, moderately activated autophagy facilitates the clearance of damaged organelles and protein aggregates. The crosstalk between exosomes and autophagy has emerged as a new therapeutic target in SCI; engineered exosomes positively regulated neuronal autophagic flux in a rat SCI model (Li RY. et al., 2023). Exosome-mediated precision delivery of paclitaxel activated mitophagy and cleared functionally exhausted mitochondria, thereby improving neuronal energy metabolism and promoting repair in an SCI model, indicating that exosomes can serve as targeted delivery carriers for autophagy inducers (Zhao et al., 2026). Dual-targeting nanoparticles enhanced microglial P2Y12 receptor expression to promote neuronal mitophagy and maintain mitochondrial quality control in an SCI model, indicating that nanoscale regulation across cell types can indirectly support neuronal mitochondrial health (Tian et al., 2026). Regarding mitochondrial replacement therapy, nanozyme-anchored mitofactories efficiently transplanted high-quality mitochondria into damaged neural cells, replaced dysfunctional mitochondria, restored oxidative phosphorylation, and rebuilt motor function in a rat SCI model, indicating that organelle-level nanoregulation represents an entirely new therapeutic paradigm (Wei et al., 2025).

### 4.5 Promotion of axonal regeneration

Promoting the regeneration of injured axons and the reconstruction of neural circuits is the fundamental route to functional recovery after SCI. Regarding the removal of extrinsic inhibition, graphene oxide-immobilized chondroitinase ABC efficiently degraded CSPGs and eliminated the chemical inhibitory barrier of the scar in a rat SCI model, indicating that nanocarriers can improve enzyme stability and delivery efficiency (Hassanli et al., 2023). Biomimetic spinal cord fiber scaffolds provided physical guidance channels for the directional growth of axons, bypassed the inhibitory microenvironment, and achieved functional reconstruction across the lesion in SCI rats, indicating that physically guided structures can compensate for the adverse chemical microenvironment (Chen et al., 2023; Usmani et al., 2020). Regarding the activation of intrinsic regenerative programs, intranasal delivery of MSC exosomes loaded with PTEN small interfering RNA reactivated regeneration-associated signaling and promoted axonal extension in a mouse SCI model, indicating that non-invasive delivery strategies can effectively activate intrinsic regenerative programs (Guo et al., 2019). The NanoScript nonviral platform achieved transient and reversible PTEN suppression and promoted axonal regeneration within a safe time window in an SCI model, indicating that nonviral nanoplatforms can safely modulate the expression of growth-inhibitory genes (Conklin et al., 2026). ROS-responsive mesoporous silica nanoparticles delivered a DUSP26 inhibitor and enhanced the intrinsic regenerative capacity of neurons by maintaining ERK phosphorylation levels in SCI rats, indicating that targeting a specific phosphatase can release the brake on regenerative signaling (Zhang et al., 2025). Sustained release of an O-GlcNAc transferase inhibitor from 3D-bioprinted scaffolds promoted the neuronal differentiation of neural stem cells and supported axonal extension in an SCI model, indicating that scaffold-mediated delivery of enzyme inhibitors can directionally determine the differentiation fate of stem cells (Liu et al., 2022a). p21 inhibition significantly enhanced the proliferation and neuronal differentiation potential of endogenous neural stem cells in an SCI model, indicating that targeted inhibition of cell cycle regulators can reactivate endogenous regenerative programs (Xiong T. et al., 2025). An exosome-collagen scaffold recruited endogenous neural stem cells through a bio-specific peptide and delivered pro-growth factors in SCI rats, indicating that combining scaffolds with exosomes can simultaneously address cell recruitment and factor delivery (Zhang L. et al., 2021). A functionalized collagen scaffold delivering miR-21-loaded exosomes significantly promoted repair in an SCI model, indicating that scaffold-mediated miRNA delivery can enhance the pro-regenerative efficacy of exosomes (Liu et al., 2022b). Regarding microtubule stabilization and the delivery of guidance factors, the long-acting pharmaceutical design of a paclitaxel-idebenone prodrug nanomedicine maintained effective concentrations of microtubule stabilizers in the lesion, promoted axonal extension, and simultaneously scavenged ROS in a rat SCI model, indicating that the prodrug strategy can balance pro-regeneration with neuroprotection (Xu et al., 2024). EGFR antibody-tethered scaffolds and cetuximab-paclitaxel co-modified collagen scaffolds blocked aberrant EGFR-ERK signaling, relieved differentiation inhibition, and significantly increased the neuronal output of endogenous neurogenesis in SCI models, indicating that functional modification of scaffold surfaces can simultaneously modulate multiple regeneration-related signals (Xu B. et al., 2017; Fan et al., 2018; Li et al., 2018; Xue et al., 2020). Epothilone D promoted the formation of relay neurons from transplanted neural stem cells through Apol8 upregulation in an SCI model, indicating that microtubule stabilizers can regulate the circuit-integration capacity of transplanted cells (Xue W. et al., 2021). Regarding synergism with the physical microenvironment, chiral nanofibers that reprogram neural stem cell metabolism, electric field-bridging scaffolds, conductive aligned fibers, and wireless magnetoelectric stimulation systems promoted neuronal differentiation and directional axonal extension through the synergy of electrical signals and topographical structure, indicating that the physical parameters of materials themselves can act as pro-regenerative driving forces (Hu et al., 2025; Han et al., 2024; Wu et al., 2025; Fontana et al., 2023; Mozhdehbakhsh Mofrad and Shamloo, 2023). A low-stress microenvironment likewise pushed neural progenitor cells toward mature neurons *in vitro*, indicating that optimization of the mechanical microenvironment is an important dimension of regeneration regulation (De Vincentiis et al., 2023). A physicochemical strategy combining electric fields with polyethylene glycol promoted the reconnection and repair of injured spinal cords in an SCI model, indicating that multimodal intervention can produce complementary effects (Zhang et al., 2022). Oriented nanotopography even overrode the inhibitory signals of the Schwann cell-astrocyte boundary itself *in vitro*, indicating that nanostructure guidance can transcend cell-level inhibition (Achenbach et al., 2023; Liu et al., 2023). Intrathecal anti-Nogo-A antibodies showed safety and some efficacy in a randomised, double-blind, multicentre, placebo-controlled phase 2b trial in patients with acute cervical SCI, providing direct evidence for the clinical feasibility of neutralizing myelin inhibitors, and nanocarrier-mediated delivery is expected to further improve the pharmacokinetic profile of such macromolecules (Weidner et al., 2025) (Figure 4).

**FIGURE 4:**
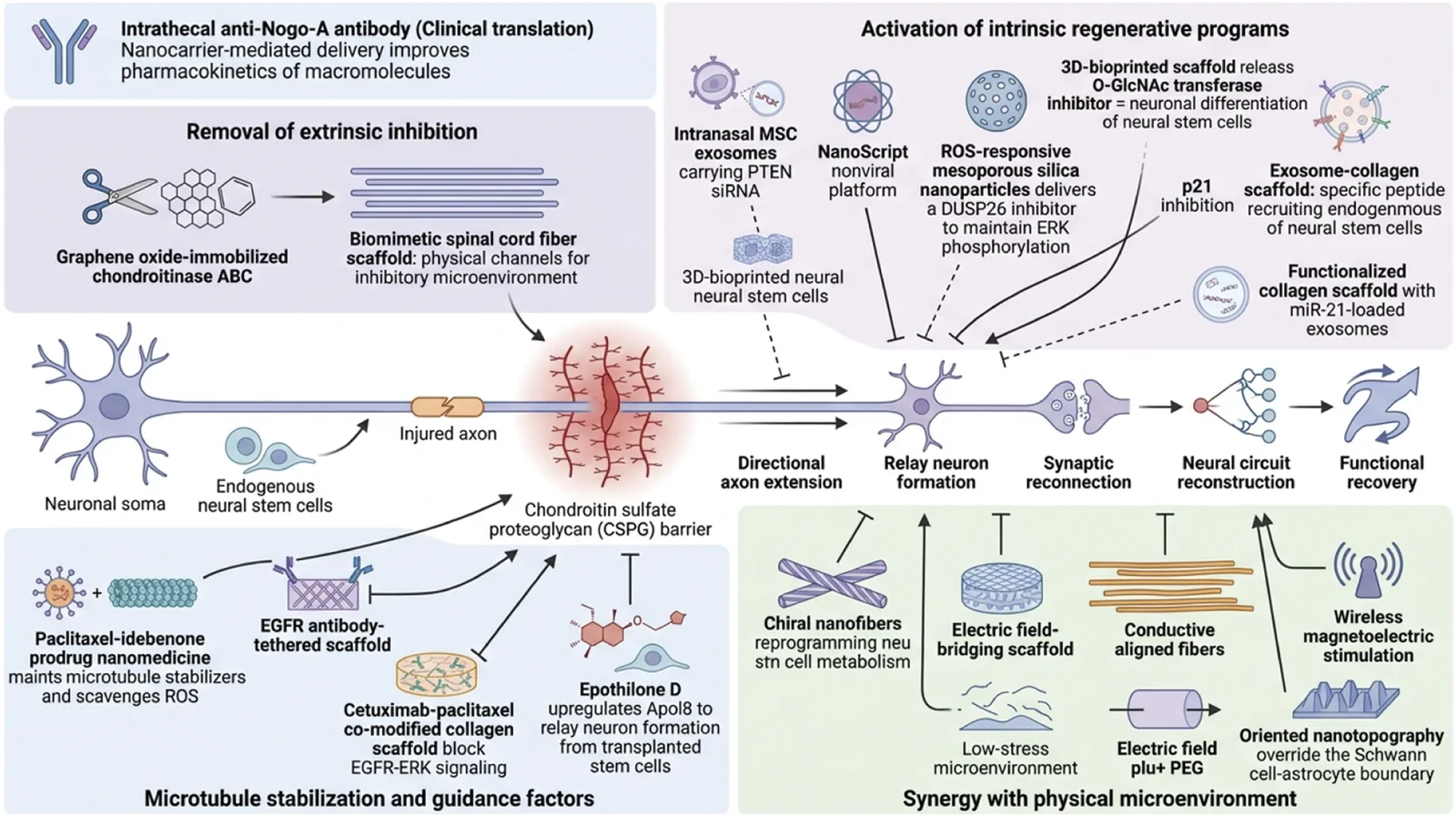
Nanocarriers and engineered scaffolds promote axon regeneration and neural circuit reconstruction after SCI. Removal of extrinsic inhibition (GO-ChABC degrades CSPG; biomimetic fibrous scaffold provides physical guidance), activation of intrinsic regenerative programs (PTEN siRNA exosomes, NanoScript, DUSP26 inhibitor, O-GlcNAc transferase inhibitor, p21 inhibition, exosome-collagen scaffold, miR-21 exosomes), microtubule stabilization/guidance factor delivery (paclitaxel-idebenone prodrug, EGFR-ERK blockade, epothilone D/Apol8), synergy with the physical microenvironment (chiral nanofibers, electric field-bridging scaffold, conductive aligned fibers, wireless magnetoelectric stimulation, low-stress microenvironment, electric field plus PEG, oriented nanotopography), and anti-Nogo-A antibody translation converge on injured axons to promote directional extension, relay neuron formation, synaptic reconnection, and functional recovery.

### 4.6 Promotion of myelin repair

Myelin repair is the key to restoring axonal conduction, and its core lies in promoting the differentiation of oligodendrocyte progenitor cells into mature oligodendrocytes as well as the synthesis and stabilization of neo-myelin.Regarding immune microenvironment remodeling in support of remyelination, the Angiopep-2-engineered exosomes described above were confirmed by single-cell sequencing to shift lesion cells toward pro-repair subtypes, significantly promoting sphingomyelin phagocytosis by microglia and macrophages and accelerating axonal remyelination and extension in a rat SCI model, indicating that engineered exosomes can simultaneously regulate immune phenotype and remyelination (Kong et al., 2025). The miR-672–5p axis described above likewise preserves myelin by suppressing inflammatory pathways in SCI rats, indicating that exosomal miRNA cargo can coordinate inflammation and myelin maintenance (Zhou et al., 2022). Regarding the targeted delivery of pro-differentiation factors and drugs, miR-124-3p-loaded mesoporous silica disguised with stem cell membranes was delivered to the lesion in an SCI model, driving microglia toward a pro-myelin-repair phenotype and improving the expression of myelin-related genes, indicating that combining gene drugs with biomimetic carriers enables the directed induction of repair phenotypes (Fan X. et al., 2024). Biomimetic spinal cord-like fiber scaffolds triggered physiological remyelination while guiding axonal regeneration in SCI rats, indicating that physical guidance structures can naturally initiate myelin reconstruction programs (Chen et al., 2023). Biomimetic zinc sulfide nanoparticles supported oligodendrocyte survival and myelin synthesis through zinc ion regulation in an SCI model, indicating that metal ion release from biomimetic carriers can support myelin anabolism (Qin et al., 2025). Regarding the clearance of myelin debris and the improvement of metabolic substrates, liposomes targeting foam-like macrophages reduced debris accumulation and restored tissue repair programs in a rat SCI model, indicating that timely debris clearance is a prerequisite for initiating oligodendrocyte progenitor differentiation (Liu Z. et al., 2025). Calcium-phytate nanoparticles synergized intracellular calcium signaling with ion storms to increase the proportion of transplanted neural stem cells differentiating into the oligodendrocyte lineage in an SCI model, indicating that ionic signaling regulation can direct the glial lineage differentiation of stem cells (Shi et al., 2026). Regarding mitochondrial functional support, mitochondrial transplantation and mitophagy activation provided energy assurance for the highly energy-demanding process of myelin synthesis, indicating that the metabolic demands of myelin repair are an important consideration in therapeutic design (Wei et al., 2025; Zhao et al., 2026).

### 4.7 Suppression of glial scar and improvement of the extracellular matrix environment

The glial scar and inhibitory extracellular matrix constitute physicochemical barriers to axonal regeneration, and nano-strategies have been developed at three levels: scar reduction, matrix modification, and structural reconstruction. Regarding scar reduction and the regulation of glial activity, a nanodrug microneedle system achieved scar-reduced repair by re-establishing microglial homeostasis in a rat SCI model, indicating that the restoration of immune homeostasis naturally mitigates scar formation (He et al., 2025). Nano-primed chemical reprogramming converted lesion glial cells into neuron-like cells *in situ* in an SCI model, replenishing neurons while suppressing glial scar formation, indicating that *in situ* reprogramming can simultaneously achieve neuronal replenishment and scar reduction (Sun X. et al., 2026). A 3D-bioprinted conductive composite hydrogel inhibited the astrocytic differentiation of neural stem cells and guided them toward neuronal differentiation, reducing scar components at their source, indicating that the physical signals of conductive materials can directionally determine stem cell differentiation (Song et al., 2023). Regarding the clearance of extracellular matrix inhibitory molecules, graphene oxide immobilization of chondroitinase ABC not only improved the thermal stability of the enzyme but also achieved sustained degradation of CSPGs in the lesion in a rat SCI model, indicating that nanocarriers can simultaneously solve the problems of enzyme stability and targeted delivery (Hassanli et al., 2023). An acellular spinal cord extracellular matrix hydrogel directly provided a natural, low-inhibitory matrix template to guide tissue regeneration in an SCI model, indicating that a natural matrix can substitute for the inhibitory components of the pathological microenvironment (Kim et al., 2026). Regarding matrix mechanics and structural reconstruction, biomimetic dual-network collagen fibers and aligned electrospun fiber-hydrogel systems remodeled the anisotropy and viscoelasticity of the matrix and provided mechanically permissive channels for axonal growth in SCI models, indicating that mechanical properties and structural anisotropy are key physical parameters guiding axonal regeneration (Zhao et al., 2024; Chen S. et al., 2022; Puhl et al., 2020). Functional hydrogels co-remodeled the migration and differentiation microenvironment and enabled endogenous neural stem cells to enter the lesion core in SCI rats, indicating that hydrogels can simultaneously address cell migration barriers and differentiation guidance (Liu D. et al., 2024; Chen Z. et al., 2022). Regarding drug penetration and delivery in the scar region, a modular egg-like hydrogel achieved individually tailored, spatiotemporally sequential drug release that progressively reprogrammed the scarred microenvironment in an SCI model, indicating that spatiotemporally controlled release can progressively overcome the scar barrier (Sun et al., 2023). A tacrolimus sustained-release collagen hydrogel combined with tissue transplantation suppressed rejection and softened the local environment in an SCI model, indicating that local sustained release of immunosuppressants can create favorable conditions for graft survival (Zhao et al., 2023). Regarding neuron-glia balance regulation, nerve growth factor-loaded anisotropic silk nanofiber hydrogels precisely tuned the ratio of neuronal to glial differentiation in an SCI model, indicating that the spatial delivery of growth factors can directionally determine cell differentiation fate (Gao et al., 2022c). Electrospun fiber scaffolds engineered glial behavior and guided glia from scar formers into supportive roles in SCI models, indicating that material topography can reprogram the functional phenotype of glial cells (Liu et al., 2023; Puhl et al., 2020).

### 4.8 Promotion of angiogenesis and blood-spinal cord barrier repair

Reconstruction of the vascular network and repair of the BSCB are prerequisites for survival and regeneration in the lesion; without timely revascularization of the hypoxic lesion core, any regenerative strategy is unlikely to succeed. Regarding the delivery of pro-angiogenic signals, M2 macrophage exosomes and hypoxia-preconditioned endothelial cell exosomes carried pro-angiogenic miRNAs and proteins, significantly increasing microvessel density and improving perfusion in the lesion in rat SCI models, indicating that exosomes can naturally deliver pro-angiogenic signals (Huang et al., 2022; Li et al., 2022). Cerebrospinal fluid-derived extracellular vesicles became enriched in pro-angiogenic signaling molecules after SCI and served as carriers of endogenous repair signals, indicating that natural vesicles in body fluids possess spontaneous repair tropism (Li C. et al., 2023). An acellular spinal cord scaffold combined with sustained VEGF release achieved persistent vascular remodeling in SCI rats, indicating that combining scaffold materials with growth factor release can provide durable vascularization signals (Xu Z. X. et al., 2017). PDGF mimetic peptide supramolecular hydrogel microspheres promoted vascularized repair through a paracrine replacement strategy in an SCI model, indicating that mimetic peptide strategies can overcome the stability problems of recombinant proteins (Wu et al., 2023). Regarding the direct replenishment of endothelial cells and tissues, transplantation of spinal cord-derived microvascular endothelial cells promoted lesion repair in an SCI model, indicating that direct supplementation of vascular endothelial cells can accelerate revascularization (You et al., 2023). Linearly oriented pre-vascularized spinal cord-like tissue engineering constructs established mature vascular networks before transplantation in an SCI model, indicating that pre-vascularization strategies can overcome the post-transplant ischemic window (Fan C. et al., 2024; Xu et al., 2023). Dual-cue scaffolds simultaneously supported neurovascular unit regeneration in an SCI model, indicating that the synchronized reconstruction of nerves and vessels is the basis of functional recovery (Liu D. et al., 2021). Regarding the protection and reconstruction of BSCB integrity, the Angiopep-2-engineered exosomes described above also maintained the structural integrity of the BSCB and reduced leakage in a rat SCI model, indicating that targeted delivery can concurrently promote neural repair and barrier protection. MMP-activatable nanodrugs crossed the disrupted BSCB and released drugs on demand in the target region in an SCI model, achieving precise delivery during the barrier-disruption window and indicating that smart delivery exploiting barrier disruption can improve targeting efficiency (Li B. et al., 2026). Laser-activated perfluorocarbon nanodroplets enabled the intrathecal delivery of macromolecular drugs through transient and reversible BSCB opening in an SCI model, indicating that physicochemical means can safely modulate barrier permeability (Nikolai et al., 2026). It should be noted that the anti-inflammatory and antioxidant strategies described above also indirectly support vascular barrier stability, as ROS scavenging by nanozymes concurrently reduces basement membrane degradation and tight junction protein loss (Shen et al., 2021; Ma et al., 2025); however, the emphasis here is on the specific mechanisms of angiogenesis and barrier repair. Angiogenesis and neurogenesis are highly coupled in space and phase, and the integrated reconstruction of the neurovascular unit is emerging as a new framework for nanotherapeutic design (Fan C. et al., 2024; Liu D. et al., 2021) (Table 3; Figure 5).

**TABLE 3:** Summary of the mechanisms of nanocarrier-based drugs in the treatment of spinal cord injury.

Mechanism	Core strategies	Representative nanocarrier systems	Experimental models	Key efficacy outcomes
Anti-inflammation and immune polarization regulation	Targeted delivery of anti-inflammatory drugs; delivery of polarization modulators; exosome-mediated reprogramming	Minocycline-dextran sulfate biomimetic liposomes; IL-4@ZIF-8 hydrogel; nanoformulated fingolimod; melatonin-primed MSC exosomes	Rat SCI models; complete transection models	TNF-alpha decreased by approximately 40%–60%; M2/M1 ratio increased 2- to 4-fold; BBB score improved by 3–6 points; lesion cavity reduced by 20%–35% (Bin et al., 2017; An et al., 2022; Liu D. et al., 2025; M Alsharbaty et al., 2026; Liu Y. et al., 2020; Liu D. et al., 2021; Xin et al., 2022)
Attenuation of oxidative stress	Nanoformulation of antioxidant enzymes; nanozyme-mediated catalytic scavenging; microenvironment-responsive release	SOD-catalase nanoparticles; cerium oxide nanozymes; Prussian blue nanozymes; ROS-responsive DUSP26 delivery	Rat SCI models	Lesional ROS levels decreased by 50%–70%; mitochondrial membrane potential restored by approximately 30%; neuronal survival increased by 20%–40% (Zhang et al., 2025; Andrabi et al., 2020; Fang and Song, 2019; Dong et al., 2020; Gao J. et al., 2024; Wang et al., 2026)
Inhibition of neuronal apoptosis	Survival pathway activation; neurotrophic factor delivery; paracrine protection	Sustained-release HGF systems; BDNF nano-delivery; melatonin-primed exosomes	Rat SCI models	Caspase-3 activation reduced by 40%–60%; TUNEL-positive cells decreased by 30%–50%; BBB score improved by 4–7 points (Yamane et al., 2019; Xia et al., 2021; Yamane et al., 2018; Andrabi et al., 2020; Liu D. et al., 2021; Zhai et al., 2021)
Regulation of programmed cell death	Ferroptosis inhibition; pyroptosis blockade; autophagy/mitophagy activation; mitochondrial transplantation	Iron-chelating microneedle patches; nanoformulated fingolimod; paclitaxel exosomes; mitofactories	Rat SCI models	GPX4 activity restored to 60%–80% of control levels; NLRP3 expression decreased by approximately 50%; mitochondrial ATP productionrecovered by approximately 40% (M Alsharbaty et al., 2026; Wei et al., 2025; Feng et al., 2025; Ding et al., 2026; Zhao et al., 2026; Tian et al., 2026)
Promotion of axonal regeneration	Disinhibition; activation of intrinsic regenerative programs; microtubule stabilization; physical guidance	Graphene oxide-chondroitinase ABC; PTEN-siRNA exosomes; paclitaxel prodrug nanoparticles; chiral nanofibers	Rat/mouse SCI models	Axon elongation length increased 2- to 5-fold; regeneration-associated genes upregulated 3- to 8-fold; BBB score improved by 4–8 points (Zhang et al., 2025; Conklin et al., 2026; Chen et al., 2023; Hassanli et al., 2023; Guo et al., 2019; Usmani et al., 2020; Wu et al., 2025)
Promotion of myelin repair	Immune microenvironment remodeling; delivery of pro-differentiation factors; debris clearance	Angiopep-2-engineered exosomes; miR-124 mesoporous silica; biomimetic spinal cord fiber scaffolds	Rat SCI models	Proportion of remyelinated axons increased 2- to 4-fold; myelin basic protein expression upregulated approximately 2-fold; conduction velocity improved by 20%–40% (Chen et al., 2023; Fan C. et al., 2024; Kong et al., 2025; Qin et al., 2025; Zhou et al., 2022; Shi et al., 2026)
Suppression of glial scar and matrix improvement	Scar reduction; matrix molecule clearance; mechanical and structural reconstruction	Nanodrug microneedles; graphene oxide-chondroitinase ABC; acellular spinal cord matrix hydrogel	Rat SCI models	Scar area reduced by 30%–50%; CSPG degradation rate of 60%–80%; axon penetration distance across the scarincreased 3- to 6-fold (Sun M. L. et al., 2026; Hassanli et al., 2023; He et al., 2025; Song et al., 2023; Kim et al., 2026)
Promotion of angiogenesis and barrier repair	Delivery of pro-angiogenic factors; endothelial replenishment; barrier protection	M2 macrophage exosomes; VEGF sustained-release scaffolds; Angiopep-2 exosomes; MMP-activatable nanodrugs	Rat SCI models	Microvessel density increased 2- to 4-fold; BSCB leakage area reduced by 40%–60%; lesion perfusion restored by approximately 50% (Li C. et al., 2026; Kong et al., 2025; Xu et al., 2017a; Huang et al., 2022; Li et al., 2022; Li C. et al., 2023; Liu W. et al., 2021)

The “Key efficacy outcomes” column reports the value ranges reported by the cited studies. Because the underlying studies differ in injury model and severity, dosing, route, timing, and endpoints, these figures are indicative of reported magnitudes rather than directly comparable across rows.

**FIGURE 5:**
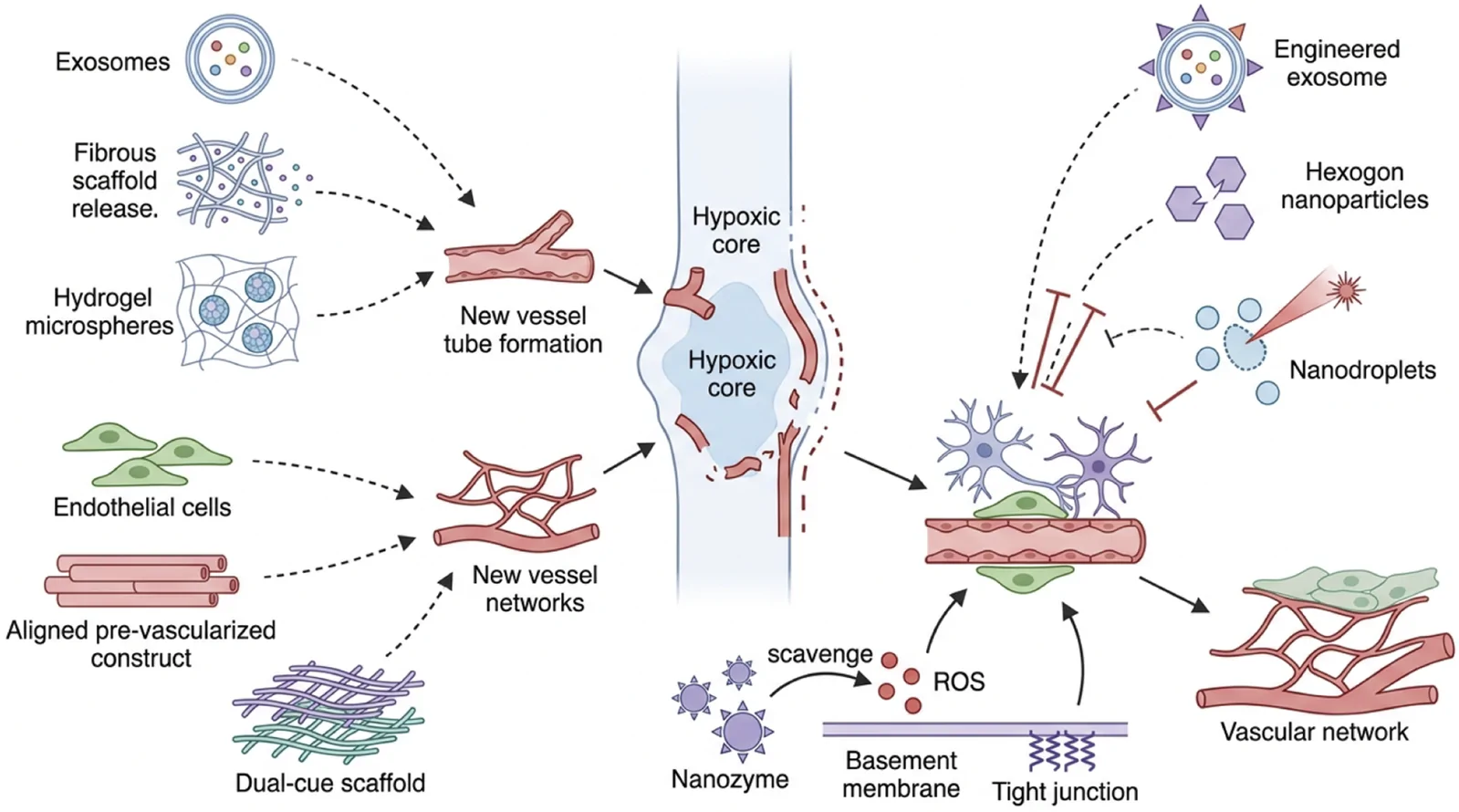
Nanocarriers and engineered scaffolds promote vascular network reconstruction and neurovascular unit integration after SCI by delivering pro-angiogenic signals, replenishing endothelial cells/pre-vascularized tissues, and protecting/rebuilding the BSCB. Exosomes, VEGF/PDGF mimetic peptides, pre-vascularized constructs, and dual-cue scaffolds enhance angiogenesis and perfusion; Angiopep-2 exosomes, MMP-activatable nanodrugs, and laser-activated nanodroplets maintain or transiently modulate the BSCB; nanozymes scavenge ROS to indirectly protect the basement membrane and tight junctions. Coupled angiogenesis and neurogenesis ultimately support lesion survival, axon regeneration, and functional recovery.

### 4.9 Stage-specific positioning and critical appraisal of current evidence

The spatiotemporally evolving pathology of SCI means that the optimal nanocarrier strategy is stage-dependent. In the acute phase, BSCB disruption, inflammatory infiltration and the oxidative burst dominate, and the transiently compromised barrier favours passive lesion accumulation, so intravenous platforms with intrinsic anti-inflammatory or antioxidant activity are most attractive. In the subacute window, when M1-to-M2 polarisation and scar maturation become therapeutically actionable, biomimetic and targeted carriers delivering polarisation modulators, neurotrophic factors or pro-regenerative miRNAs gain prominence. In the chronic phase, where cystic cavitation and a CSPG-rich scar block regeneration, scaffold-based combinatorial systems that physically bridge the lesion and provide sustained cue release become the most plausible option. This mapping is a rational inference rather than the product of stage-comparative experimentation: most studies administer the nanotherapeutic immediately after injury, whereas many patients present later, and the few delayed-treatment studies report attenuated efficacy (Guo et al., 2019; He et al., 2025).

Mechanistic conclusions in this literature rest predominantly on correlative rather than causal evidence: the co-occurrence of a molecular improvement and a functional gain is routinely interpreted as causal without loss-of-function or rescue experiments. The near-uniform reporting of positive efficacy points to publication bias, since null results and failed engineering modifications are rarely published, and genuine disagreements such as the timing-dependent dual role of inflammation are correspondingly underdiscussed. Head-to-head comparisons are almost entirely absent; nearly every study evaluates a single carrier against free-drug or untreated controls, and cross-study rankings, including the comparative judgements in Tables 2 and 4, therefore rest on inference across heterogeneous models, dosing regimens and outcome measures. Against these limitations, a pragmatic grading of translational readiness can be offered. Liposomes and polymeric nanoparticles, with the most mature manufacturing and regulatory precedent, remain the most plausible first-in-human vehicles for repurposed anti-inflammatory or neuroprotective drugs. Exosomes and nanozymes, despite promising preclinical data, are held back by manufacturing scale and chronic-toxicology uncertainties, respectively, and are unlikely to enter the clinic before the liposomal and polymeric classes. Recent systematic reviews and meta-analyses of exosome-based therapies (Su et al., 2024; Mou et al., 2025) and of antioxidant nanozymes (Lee et al., 2026; Niu et al., 2026; Ma et al., 2025) corroborate the stage-specific rationale above while confirming the same methodological weaknesses of heterogeneity, publication bias and limited rescue experimentation.

**TABLE 4:** Comparative evaluation of major nanocarrier platforms for spinal cord injury therapy.

Platform	Targeting capability	Drug loading and release	Safety profile	Typical administration	Scalability and GMP readiness	Translational potential
Liposomes	Moderate; enhanced by ligand conjugation or biomimetic coating	Encapsulation of hydrophilic and lipophilic drugs; release over hours to days	Good; extensive clinical precedent in other indications	Intravenous; intrathecal	High; established GMP processes	Near term (repurposed drugs, acute SCI)
Polymeric nanoparticles	Moderate to high; ligand-conjugatable, stimuli-responsive release	Loading usually below 10%; sustained release over days to weeks	Generally acceptable; acidic PLGA degradation products a local concern	Intravenous; local; intrathecal	High; established processes	Near term
Polymeric micelles	Low to moderate; limited by circulation stability	High solubilization of hydrophobic drugs; risk of dilution-induced disassembly	Acceptable; excipient history varies by polymer	Intravenous	Moderate	Medium term
Inorganic nanozymes and inorganic nanomaterials	Mostly passive; catalytic activity independent of payload	High effective activity through catalysis; payload capacity variable	Unresolved: long-term organ accumulation; chronic safety thresholds not established	Intravenous; local	Moderate; strongly material-dependent	Long term, pending chronic safety data
Exosomes and biomimetic carriers	High; natural tropism, further enhanced by engineering	Cargo determined by source cells; loading low and variable	Low immunogenicity; risks depend on the pathological state of source cells	Intravenous; intranasal; local	Low: yield, potency assays, and batch consistency unresolved	Medium to long term, pending GMP solutions
Nanohydrogels and composite scaffolds	Local; site-directed by implantation	High-capacity depots; release over weeks to months	Generally good; surgery-associated infection risk	Surgical implantation or injection	Moderate; patient-specific fabrication challenging	Long term (structural reconstruction in severe/chronic SCI)

## 5 Limitations of nanocarrier-based drugs for spinal cord injury

Insufficient validation of long-term safety remains the most prominent bottleneck: systematic data are lacking on nanomaterial accumulation in the spinal cord and systemic tissues, the toxicity of degradation products, and delayed effects. Nanoformulation can alter the distribution and toxicity profile of a drug while enhancing its efficacy (Gao et al., 2022a); inorganic carriers can both eliminate and introduce toxicity depending on formulation (Salvi et al., 2024); and the long-term neural safety of two-dimensional materials such as graphene remains controversial (Girão et al., 2022). Although exosomes and biomimetic carriers are comparatively well tolerated, the pathological state of their cells of origin may carry pathogenic signalling molecules that current isolation procedures cannot fully remove (Lai et al., 2022; Li Q. et al., 2026).

Most fundamentally, in contrast to the anti-Nogo-A antibody programme that has completed a multicentre phase 2b trial in acute cervical SCI (Weidner et al., 2025; Farner et al., 2026), no nanocarrier-based therapy for SCI has yet entered clinical trials, and the underlying reasons are carrier-class-specific. For inorganic nanozymes, the principal barrier is the unresolved long-term accumulation of cerium, zinc and other metal ions, for which no chronic safety threshold has been established (Singh et al., 2026; Fang and Song, 2019). For exosomes and biomimetic carriers, the bottleneck is manufacturing: yields are low, potency assays are not standardised, and batch-to-batch consistency of cargo and membrane protein orientation has not been demonstrated at GMP scale (Lai et al., 2022; Li Q. et al., 2026). For liposomes and polymeric nanoparticles, manufacturing maturity is not the constraint; the barriers are the modest efficacy margins observed preclinically, the small and heterogeneous patient population that complicates trial powering and endpoint selection, and limited commercial incentive. Repurposed-drug liposomal or polymeric formulations in acute SCI are therefore the nearest-term translational candidates, whereas exosome and nanozyme platforms require maturation of potency assays and chronic toxicology, respectively, before first-in-human testing becomes defensible. From a regulatory and manufacturing standpoint, no indication-specific framework yet exists for nanomedicines targeting the CNS, although approved nanosystems in other fields show that the pathway is navigable when supported by extensive biodistribution, immunogenicity and manufacturing data. Particle size distribution, zeta potential, ligand density, drug-to-carrier ratio and, for vesicles, cargo potency and membrane protein orientation must be defined as critical quality attributes with validated assays before GMP production can begin (Lai et al., 2022; Li Q. et al., 2026). Repeated-dose immunogenicity deserves particular attention, because complement activation-related pseudoallergy has been documented for intravenous nanosystems and chronically treated SCI patients would face cumulative exposure (Zhang Z. et al., 2026; Kartha et al., 2020; Xiao et al., 2025). A consensus minimum dataset covering biodistribution, GLP-compliant chronic toxicology and standardised potency assays, agreed by investigators, manufacturers and regulators, is the precondition for first-in-human SCI nanomedicine trials (Lai et al., 2022; Li Q. et al., 2026; Salvi et al., 2024). Incomplete understanding of *in vivo* metabolism and clearance likewise constrains translation. The intrathecal metabolic fate, degradation kinetics and redistribution of most carriers lack quantitative characterisation, and organ accumulation of cerium-, selenium-, manganese- and zinc-based nanoparticles still requires long-term toxicological support (Singh et al., 2026; Fang and Song, 2019). Targeting efficiency is limited by protein-corona inactivation of surface ligands, elevated interstitial pressure that weakens penetration into the lesion core, and rapid BSCB remodelling that narrows the permeability window (Li B. et al., 2026; Ma et al., 2022), so overall targeted delivery efficiency remains generally low despite stimuli-responsive designs (Li B. et al., 2026; Zhuang et al., 2025).

Translating animal results to the clinic is the central obstacle: rodent hemisection or contusion models differ markedly from the heterogeneous mixed pathology of human SCI, and the high proportion of spontaneous recovery in rodents readily leads to efficacy overestimation (Khan et al., 2020; Gu et al., 2024). Most preclinical dosing occurs immediately after injury, whereas clinical patients typically miss this window, and limited batch-to-batch stability of multifunctional nanozymes, engineered exosomes and 3D-printed scaffolds further constrains scale-up (Li Q. et al., 2026; Sun et al., 2023).

Finally, the lack of unified evaluation standards is an institutional bottleneck: wide differences in carrier characterisation, animal models, routes, endpoints and follow-up periods preclude horizontal comparison and meta-analysis, and a dedicated framework for spinal-cord nanomedicines is still missing, although the EV field offers templates such as MIFlowCyt and cross-species characterisation studies (Lai et al., 2022). Cost-effectiveness and intellectual-property barriers are practically significant, and given the heterogeneity of SCI, most current nano-formulations remain generic rather than individualised, although modular hydrogels demonstrate the feasibility of personalised delivery (Sun et al., 2023). Long-term safety and efficacy for chronic complications such as neuropathic pain and neurogenic bladder also require dedicated evaluation (Zhang Z. et al., 2026; Kartha et al., 2020; Xiao et al., 2025).

## 6 Conclusion

This review has systematically summarized the research progress of nanocarrier-based therapeutics for SCI and revealed that nanocarriers exert therapeutic effects through seven major mechanisms. At the level of inflammation and immune regulation, nanocarriers achieve spatiotemporal control of inflammatory cascades through the targeted delivery of anti-inflammatory drugs and the regulation of M1/M2 polarization of macrophages and microglia. At the level of oxidative stress antagonism, the nanoformulation of antioxidant enzymes, nanozyme-mediated catalytic scavenging, and microenvironment-responsive release constitute three complementary strategies. At the level of cell death regulation, precise intervention has continuously deepened from classical apoptosis inhibition to multiple regulated death modalities including ferroptosis, pyroptosis, and autophagy. At the level of axonal regeneration, the multidimensional synergy of disinhibition, activation of intrinsic regenerative programs, and microtubule stabilization combined with physical guidance provides new routes for neural circuit reconstruction. At the level of myelin repair, immune microenvironment remodeling and the targeted delivery of pro-differentiation factors are driving myelin regeneration from a passive bystander endpoint toward an actively designed target. At the level of glial scar management, a paradigm shift from passively avoiding the scar to actively remodeling it is underway. At the level of vascular repair, the synchronized advancement of pro-angiogenic factor delivery with BSCB protection and reconstruction lays the foundation for the integrated rebuilding of the neurovascular unit.

This review finds that liposomes, polymeric nanoparticles, polymeric micelles, inorganic nanomaterials, exosomes and biomimetic carriers, as well as nanohydrogels and composite scaffolds, each have distinct advantages. Among the six carrier categories, biomimetic nanocarriers and exosomes appear, on current preclinical evidence, to hold comparatively favorable near-term prospects for clinical translation owing to their combined low immunogenicity and natural targeting ability; multifunctional nanozymes may represent the direction of medium- and long-term technological breakthroughs by virtue of their sustained catalytic activity and theranostic potential; and nanohydrogels and composite scaffolds have irreplaceable structural repair value in severe injury scenarios such as complete SCI. In terms of the delivered therapeutics, anti-inflammatory drugs such as glucocorticoids and minocycline, immunomodulators such as fingolimod, microtubule stabilizers such as paclitaxel, natural polyphenols such as curcumin, neurotrophic factors and nucleic acid drugs, together with endogenously active nanozymes and gasotransmitter donors, constitute the core armamentarium of nanocarrier-based therapeutics.

Looking forward, the development of nanocarrier-based therapeutics will show three trends. In terms of intelligence, microenvironment-responsive release, theranostic integration, and closed-loop control designs will further improve the spatiotemporal precision of therapy; meanwhile, the development of microstructure-based sensors such as flexible pressure sensors and wearable multimodal sensing and feedback systems will provide new tools for the objective, quantitative assessment of motor function recovery after SCI, compensating for the subjectivity and insufficient sensitivity of traditional behavioral scores (Yang et al., 2019; Kang et al., 2025). In terms of systematization, the deep integration of nanocarriers with stem cells, gene editing, and neuromodulation technologies will enable the integrated reconstruction of the injury microenvironment at the molecular, cellular, and circuit levels. In terms of standardization, unified quality evaluation standards, scalable preparation processes, and standardized clinical trial design constitute the obligatory path across the translational divide. Although bottlenecks in long-term safety, targeting efficiency, batch stability, and clinical translation persist, with the deepening cross-fertilization of materials science, neurobiology, and regenerative medicine, nanocarrier-based drugs are expected to become an important component of comprehensive SCI therapy and ultimately benefit the large population of patients with SCI.

## References

[B1] Achenbach P. Hillerbrand L. Gerardo-Nava J. L. Dievernich A. Hodde D. Sechi A. S. (2023). Function follows form: oriented substrate nanotopography overrides neurite-repulsive Schwann cell-astrocyte barrier formation in an *in vitro* model of glial scarring. Nano Lett. 23 (14), 6337–6346. 10.1021/acs.nanolett.3c00873 37459449

[B2] Agrawal L. Vimal S. K. Barzaghi P. Shiga T. Terenzio M. (2022). Biodegradable and electrically conductive melanin-poly (3-Hydroxybutyrate) 3D fibrous scaffolds for neural tissue engineering applications. Macromol. Biosci. 22 (12), e2200315. 10.1002/mabi.202200315 36114714

[B3] Ahmadpour Y. Bahrami G. Arkan E. Abbaszadeh F. Aghaz F. Fakhri S. (2025). Unveiling the effects of Rosa canina oligosaccharide liposome on neuropathic pain and motor dysfunction following spinal cord injury in rats: relevance to its antioxidative effects. Front. Pharmacol. 16, 1533025. 10.3389/fphar.2025.1533025 PMC11868053 40028155

[B4] An J. Jiang X. Wang Z. Li Y. Zou Z. Wu Q. (2022). Codelivery of minocycline hydrochloride and dextran sulfate *via* bionic liposomes for the treatment of spinal cord injury. Int. J. Pharm. 628, 122285. 10.1016/j.ijpharm.2022.122285 36244559

[B5] Andrabi S. S. Yang J. Gao Y. Kuang Y. Labhasetwar V. (2020). Nanoparticles with antioxidant enzymes protect injured spinal cord from neuronal cell apoptosis by attenuating mitochondrial dysfunction. J. Control Release 317, 300–311. 10.1016/j.jconrel.2019.12.001 31805339 PMC7007870

[B6] Bai B. Wang J. Jiang L. Zou C. Liu S. Qi Z. (2026). Neonatal spinal-cord-like scaffold with hierarchical structural and neurogenetic microenvironments for spinal cord injury repair. ACS Nano 20 (7), 5423–5437. 10.1021/acsnano.5c07071 41671503

[B7] Behroozi Z. (#) Rahimi B. (#) Motamednezhad A. Ghadaksaz A. Hormozi-Moghaddam Z. Moshiri A. (2024). Combined effect of cerium oxide nanoparticles loaded scaffold and photobiomodulation therapy on pain and neuronal regeneration following spinal cord injury: an experimental study. Photochem Photobiol. Sci. 23 (2), 225–243. 10.1007/s43630-023-00501-6 38300466

[B8] Bin S. Zhou N. Pan J. Pan F. Wu X. F. Zhou Z. H. (2017). Nano-carrier mediated co-delivery of methyl prednisolone and minocycline for improved post-traumatic spinal cord injury conditions in rats. Drug Dev. Ind. Pharm. 43 (6), 1033–1041. 10.1080/03639045.2017.1291669 28279078

[B9] Boyko A. Ksenofontov A. Ryabov S. Baratova L. Graf A. Bunik V. (2018). Delayed influence of spinal cord injury on the amino acids of NO(•) metabolism in rat cerebral cortex is attenuated by thiamine. Front. Med. (Lausanne) 4, 249. 10.3389/fmed.2017.00249 29379782 PMC5775235

[B10] Boyko A. Tsepkova P. Aleshin V. Artiukhov A. Mkrtchyan G. Ksenofontov A. (2021). Severe spinal cord injury in rats induces chronic changes in the spinal cord and cerebral cortex metabolism, adjusted by thiamine that improves locomotor performance. Front. Mol. Neurosci. 14, 620593. 10.3389/fnmol.2021.620593 PMC8044794 33867932

[B11] Boyko A. I. Karlina I. S. Zavileyskiy L. G. Aleshin V. A. Artiukhov A. V. Kaehne T. (2022). Delayed impact of 2-Oxoadipate dehydrogenase inhibition on the rat brain metabolism is linked to protein glutarylation. Front. Med. (Lausanne) 9, 896263. 10.3389/fmed.2022.896263 PMC9198357 35721081

[B12] Cao X. Li X. Shi W. Adu-Frimpong M. Li J. Huang H. (2025). 3D-Bioprinted scaffolds with precise factor delivery for spinal cord injury repair. ACS Appl. Mater Interfaces 17 (30), 42586–42601. 10.1021/acsami.5c03076 40673827

[B13] Chen S. Wu C. Zhou T. Wu K. Xin N. Liu X. (2022a). Aldehyde-methacrylate-hyaluronan profited hydrogel system integrating aligned and viscoelastic cues for neurogenesis. Carbohydr. Polym. 278, 118961. 10.1016/j.carbpol.2021.118961 34973776

[B14] Chen Z. Zhang H. Fan C. Zhuang Y. Yang W. Chen Y. (2022b). Adhesive, stretchable, and spatiotemporal delivery fibrous hydrogels harness endogenous neural stem/progenitor cells for spinal cord injury repair. ACS Nano 16 (2), 1986–1998. 10.1021/acsnano.1c06892 34842412

[B15] Chen Z. Sun Z. Fan Y. Yin M. Jin C. Guo B. (2023). Mimicked spinal cord fibers trigger axonal regeneration and remyelination after injury. ACS Nano 17 (24), 25591–25613. 10.1021/acsnano.3c09892 38078771

[B16] Chen T. Zhang Q. Zhang L. Ai Y. Chen Z. Liu J. (2025). Induced pluripotent stem cell-derived extracellular vesicles prevent neural stem cell senescence to promote cognitive recovery after traumatic brain injury. ACS Nano 19 (43), 37783–37801. 10.1021/acsnano.5c10672 41128616

[B17] Cheng Y. Wang R. Bai Z. Han L. Zhu X. Li M. (2026). ROS-responsive trifluoperazine prodrug nanoparticles and mesenchymal stem cell exosomes synergistically modulate astrocyte phenotype for spinal cord injury repair. ACS Appl. Mater Interfaces 18 (20), 28587–28599. 10.1021/acsami.6c09304 PMC13220221 42131986

[B18] Cheshmi H. Mohammadi H. Akbari M. Nasiry D. Rezapour-Nasrabad R. Bagheri M. (2023). Human placental mesenchymal stem cell-derived exosomes in combination with hyperbaric oxygen synergistically promote recovery after spinal cord injury in rats. Neurotox. Res. 41 (5), 431–445. 10.1007/s12640-023-00649-0 37155125

[B19] Conklin B. Liu Y. Nevins S. Song B. G. Chueng S. T. D. Xiaowen Q. (2026). NanoScript-Enabled nonviral transient repression of phosphatase and tensin homolog for axonal regeneration and central nervous system injury repair. ACS Nano 20 (8), 6582–6608. 10.1021/acsnano.5c13020 PMC12961949 41711373

[B20] De Vincentiis S. Baggiani M. Merighi F. Cappello V. Lopane J. Di Caprio M. (2023). Low forces push the maturation of neural precursors into neurons. Small 19 (30), e2205871. 10.1002/smll.202205871 37058009

[B21] Ding Y. Chen Y. Tao Y. Zhang Z. Ge M. Shi J. (2026). An iron-scavenging and hydrogen-releasing microneedle patch suppresses ferroptosis and promotes spinal cord repair. ACS Nano 20 (16), 12210–12226. 10.1021/acsnano.5c19206 41960786

[B22] Dong L. Kang X. Ma Q. Xu Z. Sun H. Hao D. (2020). Novel approach for efficient recovery for spinal cord injury repair *via* biofabricated nano-cerium oxide loaded PCL with resveratrol to improve *in vitro* biocompatibility and autorecovery abilities. Dose Response 18 (3), 1559325820933518. 10.1177/1559325820933518 32952482 PMC7476352

[B23] Fan C. Li X. Zhao Y. Xiao Z. Xue W. Sun J. (2018). Cetuximab and taxol co-modified collagen scaffolds show combination effects for the repair of acute spinal cord injury. Biomater. Sci. 6 (7), 1723–1734. 10.1039/c8bm00363g 29845137

[B24] Fan X. Shi L. Yang Z. Li Y. Zhang C. Bai B. (2024). Targeted repair of spinal cord injury based on miRNA-124-3p-Loaded mesoporous silica camouflaged by stem cell membrane modified with rabies virus glycoprotein. Adv. Sci. (Weinh) 11 (21), e2309305. 10.1002/advs.202309305 38509833 PMC11151008

[B25] Fan C. Cai H. Zhang L. Wu X. Yan J. Jin L. (2024). Constructing linear-oriented pre-vascularized human spinal cord tissues for spinal cord injury repair. Adv. Healthc. Mater 13 (18), e2303388. 10.1002/adhm.202303388 38537119

[B26] Fang X. Song H. (2019). Synthesis of cerium oxide nanoparticles loaded on chitosan for enhanced auto-catalytic regenerative ability and biocompatibility for the spinal cord injury repair. J. Photochem Photobiol. B 191, 83–87. 10.1016/j.jphotobiol.2018.11.016 30594737

[B27] Farner L. Scheuren P. S. Sharifi K. Emmenegger T. M. Seif M. Hubli M. (2026). Anti-Nogo-A NG101 treatment induces changes in spinal cord micro- and macrostructure following spinal cord injury. Nat. Commun. 17 (1), 4197. 10.1038/s41467-026-71412-0 42120375 PMC13168246

[B28] Feng C. Wang Q. Xiao S. Yang Y. Chen B. Li H. (2025). Adaptive phenol-based hydrogel embedded with metformin nanoparticles targets oxidative stress and ferroptosis for spinal cord injury repair. ACS Nano 19 (38), 33960–33980. 10.1021/acsnano.5c09794 40973102

[B29] Fontana S. Caramazza L. Marracino P. Cuenca Ortolá I. Colella M. Dolciotti N. (2023). Electric field bridging-effect in electrified microfibrils' scaffolds. Front. Bioeng. Biotechnol. 11, 1264406. 10.3389/fbioe.2023.1264406 PMC10634785 37954020

[B30] Gao X. Hassan M. M. Ghosh S. Mao G. Sankari A. (2022a). Efficacy and toxicity of the DPCPX nanoconjugate drug study for the treatment of spinal cord injury in rats. J. Appl. Physiol. 133 (2), 262–272. 10.1152/japplphysiol.00195.2022 PMC9342139 35771225

[B31] Gao X. Han Z. Huang C. Lei H. Li G. Chen L. (2022b). An anti-inflammatory and neuroprotective biomimetic nanoplatform for repairing spinal cord injury. Bioact. Mater 18, 569–582. 10.1016/j.bioactmat.2022.05.026 PMC9256979 35845318

[B32] Gao X. Cheng W. Zhang X. Zhou Z. Ding Z. Zhou X. (2022c). Nerve growth factor-laden anisotropic silk nanofiber hydrogels to regulate neuronal/astroglial differentiation for scarless spinal cord repair. ACS Appl. Mater Interfaces 14 (3), 3701–3715. 10.1021/acsami.1c19229 35006667

[B33] Gao J. Chen S. Lin S. Mei X. (2024). Prussian blue analogues improves the microenvironment after spinal cord injury by regulating Zn. Int. Immunopharmacol. 131, 111868. 10.1016/j.intimp.2024.111868 38493692

[B34] Gao X Jin B. (#) Zhou X. Bai J. Zhong H. Zhao K. (2024). Recent advances in the application of gasotransmitters in spinal cord injury. J. Nanobiotechnology 22 (1), 277. 10.1186/s12951-024-02523-3 38783332 PMC11112916

[B35] Girão A. F. Serrano M. C. Completo A. Marques P. A. A. P. (2022). Is graphene shortening the path toward spinal cord regeneration? ACS Nano 16 (9), 13430–13467. 10.1021/acsnano.2c04756 36000717 PMC9776589

[B36] Gu X. (#) Zhang S. (#) Ma W. (2024). Bibliometric analysis of nanotechnology in spinal cord injury: current status and emerging frontiers. Front. Pharmacol. 15, 1473599. 10.3389/fphar.2024.1473599 39723251 PMC11668783

[B37] Guo S. Perets N. Betzer O. Ben-Shaul S. Sheinin A. Michaelevski I. (2019). Intranasal delivery of mesenchymal stem cell derived exosomes loaded with phosphatase and tensin homolog siRNA repairs complete spinal cord injury. ACS Nano 13 (9), 10015–10028. 10.1021/acsnano.9b01892 31454225

[B38] Han S. Xiao Z. Li X. Zhao H. Wang B. Qiu Z. (2018). Human placenta-derived mesenchymal stem cells loaded on linear ordered collagen scaffold improves functional recovery after completely transected spinal cord injury in canine. Sci. China Life Sci. 61 (1), 2–13. 10.1007/s11427-016-9002-6 28527111

[B39] Han M. Yang H. Lu X. Li Y. Liu Z. Li F. (2022). Three-dimensional-cultured MSC-derived exosome-hydrogel hybrid microneedle array patch for spinal cord repair. Nano Lett. 22 (15), 6391–6401. 10.1021/acs.nanolett.2c02259 35876503

[B40] Han S. Zhang D. Kao Y. Zhou X. Guo X. Zhang W. (2024). Trojan horse strategy for wireless electrical stimulation-induced Zn(2+) release to regulate neural stem cell differentiation for spinal cord injury repair. ACS Nano 18 (47), 32517–32533. 10.1021/acsnano.4c08863 39527695

[B41] Harrell C. R. Volarevic V. Djonov V. Volarevic A. (2022). Therapeutic potential of exosomes derived from adipose tissue-sourced mesenchymal stem cells in the treatment of neural and retinal diseases. Int. J. Mol. Sci. 23 (9), 4487. 10.3390/ijms23094487 35562878 PMC9105552

[B42] Hassanli A. Daneshjou S. Dabirmanesh B. Khajeh K. (2023). Improvement of thermal-stability of chondroitinase ABCI immobilized on graphene oxide for the repair of spinal cord injury. Sci. Rep. 13 (1), 18220. 10.1038/s41598-023-45555-9 37880390 PMC10600109

[B43] He W. Zhang L. Zheng D. Tang L. Zhang Y. Peng M. (2025). Nanodrug-loaded microneedles promote scar-reduced repair after spinal cord injury by re-establishing microglial homeostasis. Acta Biomater. 198, 440–451. 10.1016/j.actbio.2025.04.026 40222602

[B44] Hu Y. Zhao X. Wang W. Zhang Z. Wang L. Lu X. (2025). MXene film-mediated wireless magnetoelectric stimulation to promote neuronal differentiation in spinal cord injury neural stem cell therapy. ACS Nano 19 (39), 34643–34658. 10.1021/acsnano.5c08287 40982631

[B45] Hu F. Zheng G. Li Y. Wu M. Fu J. Wang Z. (2026). Injectable metal-free carbon dot-integrated hydrogel for regulating inflammation *via* macrophage metabolic reprograming in spinal cord injury therapy. ACS Nano 20 (12), 9603–9618. 10.1021/acsnano.5c13627 41830797

[B46] Huang J. H. He H. Chen Y. N. Liu Z. Romani M. D. Xu Z. Y. (2022). Exosomes derived from M2 macrophages improve angiogenesis and functional recovery after spinal cord injury through HIF-1α/VEGF axis. Brain Sci. 12 (10), 1322. 10.3390/brainsci12101322 36291255 PMC9599527

[B47] Huang T. Wu J. Mu J. Gao J. (2023). Advanced therapies for traumatic central nervous system injury: delivery strategy reinforced efficient microglial manipulation. Mol. Pharm. 20 (1), 41–56. 10.1021/acs.molpharmaceut.2c00605 36469398

[B48] Ji Z. Yan J. Li C. Sun Y. Wang S. Tao J. (2025). Precise rewiring of corticospinal axons and spinal interneurons *via* near-infrared optogenetics for spinal cord injury treatment. Sci. Adv. 11 (31), eads4938. 10.1126/sciadv.ads4938 40749050 PMC12315976

[B49] Jia Y. (#) Wang J. Zhang T. Zhu S. Wang N. He Q. (2026). Spinal cord stimulation for concurrent improvement of consciousness and spasticity following severe traumatic brain injury: a case study. Front. Neurosci. 20, 1866882. 10.3389/fnins.2026.1866882 PMC13458046 42582668

[B50] Ju Y. Hu Y. Yang P. Xie X. Fang B. (2022). Extracellular vesicle-loaded hydrogels for tissue repair and regeneration. Mater Today Bio 18, 100522. 10.1016/j.mtbio.2022.100522 36593913 PMC9803958

[B51] Kang T. W. Lee Y. J. Rigo B. Soltis I. Lee J. Kim H. (2025). Soft nanomembrane sensor-enabled wearable multimodal sensing and feedback system for upper-limb sensory impairment assistance. ACS Nano 19 (5), 5613–5628. 10.1021/acsnano.4c15530 39888714 PMC11823636

[B52] Kartha S. Yan L. Ita M. E. Amirshaghaghi A. Luo L. Wei Y. (2020). Phospholipase A(2) inhibitor-loaded phospholipid micelles abolish neuropathic pain. ACS Nano 14 (7), 8103–8115. 10.1021/acsnano.0c00999 32484651 PMC7438274

[B53] Khaledian S. Mohammadi G. Abdoli M. Fatahian A. Fatahian A. Fatahian R. (2024). Recent advances in implantable 3D-Printed scaffolds for repair of spinal cord injury. Adv. Pharm. Bull. 14 (2), 331–345. 10.34172/apb.2024.032 39206398 PMC11347741

[B54] Khan T. I. Hemalatha S. Waseem M. (2020). Promising role of nano-encapsulated drugs for spinal cord injury. Mol. Neurobiol. 57 (4), 1978–1985. 10.1007/s12035-019-01862-9 31900861

[B55] Kim H. Y. Kumar H. Jo M. J. Kim J. Yoon J. K. Lee J. R. (2018). Therapeutic efficacy-potentiated and diseased organ-targeting nanovesicles derived from mesenchymal stem cells for spinal cord injury treatment. Nano Lett. 18 (8), 4965–4975. 10.1021/acs.nanolett.8b01816 29995418

[B56] Kim J. Zhang S. Jung J. H. Lee M. J. Han I. Cho S. W. (2026). Spinal cord extracellular matrix hydrogel enhances organoid maturation and functional regeneration after spinal cord injury. Mater Today Bio 38, 103168. 10.1016/j.mtbio.2026.103168 42111758 PMC13153651

[B57] Kong G. Liu J. Wang J. Yu X. Li C. Deng M. (2025). Engineered extracellular vesicles modified by Angiopep-2 peptide promote targeted repair of spinal cord injury and brain inflammation. ACS Nano 19 (4), 4582–4600. 10.1021/acsnano.4c14675 PMC11803916 39853366

[B58] Krupa P. Svobodova B. Dubisova J. Kubinova S. Jendelova P. Machova Urdzikova L. (2019). Nano-formulated curcumin (lipodisq™) modulates the local inflammatory response, reduces glial scar and preserves the white matter after spinal cord injury in rats. Neuropharmacology 155, 54–64. 10.1016/j.neuropharm.2019.05.018 31108112

[B59] Lai J. J. Chau Z. L. Chen S. Y. Hill J. J. Korpany K. V. Liang N. (2022). Exosome processing and characterization approaches for research and technology development. Adv. Sci. (Weinh) 9 (15), e2103222. 10.1002/advs.202103222 35332686 PMC9130923

[B60] Lee J. M. Kim H. O. Byun S. W. Kim Y. J. Singh M. K. Kim S. S. (2026). Reprogramming the oxidative microenvironment: antioxidant nanoplatforms for central nervous system repair. Mater Today Bio 40, 103543. 10.1016/j.mtbio.2026.103543 42621313 PMC13487319

[B61] Li X. Fan C. Xiao Z. Zhao Y. Zhang H. Sun J. (2018). A collagen microchannel scaffold carrying paclitaxel-liposomes induces neuronal differentiation of neural stem cells through Wnt/β-catenin signaling for spinal cord injury repair. Biomaterials 183, 114–127. 10.1016/j.biomaterials.2018.08.037 30153562

[B62] Li L. Xiao B. Mu J. Zhang Y. Zhang C. Cao H. (2019). A MnO(2) nanoparticle-dotted hydrogel promotes spinal cord repair *via* regulating reactive oxygen species microenvironment and synergizing with mesenchymal stem cells. ACS Nano 13 (12), 14283–14293. 10.1021/acsnano.9b07598 31769966

[B63] Li L. Mu J. Zhang Y. Zhang C. Ma T. Chen L. (2022). Stimulation by exosomes from hypoxia preconditioned human umbilical vein endothelial cells facilitates mesenchymal stem cells angiogenic function for spinal cord repair. ACS Nano 16 (7), 10811–10823. 10.1021/acsnano.2c02898 35786851

[B64] Li R. Y. Hu Q. Shi X. Luo Z. y. Shao D. h. (2023). Crosstalk between exosomes and autophagy in spinal cord injury: fresh positive target for therapeutic application. Cell. Tissue Res. 391 (1), 1–17. 10.1007/s00441-022-03699-6 36380098 PMC9839811

[B65] Li C. Qin T. Jin Y. Hu J. Yuan F. Cao Y. (2023). Cerebrospinal fluid-derived extracellular vesicles after spinal cord injury promote vascular regeneration *via* PI3K/AKT signaling pathway. J. Orthop. Transl. 39, 124–134. 10.1016/j.jot.2023.02.001 PMC9999163 36909861

[B66] Li Z. Yu H. Wang Z. Duan H. Li M. Liao J. (2025). Recent advances in nanotechnology for repairing spinal cord injuries. Biomaterials 323, 123422. 10.1016/j.biomaterials.2025.123422 40403446

[B67] Li B. Li T. Zhang Q. Chen L. Shuai X. Liu B. (2026). Matrix metalloproteinase-activatable nanodrug crossing blood-spinal cord barrier for On-Demand drug release in spinal cord injury repair. ACS Nano 20 (10), 8746–8761. 10.1021/acsnano.5c21926 41782502

[B68] Li Q. Ding Y. Shi Y. Qiu C. Lei L. Li S. (2026). 80 years of extracellular vesicles: from discovery to clinical translation. Extracell. Vesicles Circ. Nucl. Acids 7 (1), 165–233. 10.20517/evcna.2025.161 41695594 PMC12902917

[B69] Li C. Cheng Z. He Y. Ma C. Sun Y. Han W. (2026). Nanozyme-switched efferocytosis initiation platform orchestrates pathological network reprogramming to promote functional recovery after spinal cord injury. ACS Nano 20 (20), 14890–14909. 10.1021/acsnano.6c05701 42120967

[B70] Lin D. Lee J. M. Wang C. Park H. G. Lieber C. M. (2023). Injectable ventral spinal stimulator evokes programmable and biomimetic hindlimb motion. Nano Lett. 23 (13), 6184–6192. 10.1021/acs.nanolett.3c01806 37338198

[B71] Liu Y. Liu Y. Zang J. Abdullah A. A. I. Dong H. (2020). Design strategies and applications of ROS-responsive phenylborate ester-based nanomedicine. ACS Biomater. Sci. Eng. 6 (12), 6510–6527. 10.1021/acsbiomaterials.0c01190 33320631

[B72] Liu W. Rong Y. Wang J. Zhou Z. Ge X. Ji C. (2020). Exosome-shuttled miR-216a-5p from hypoxic preconditioned mesenchymal stem cells repair traumatic spinal cord injury by shifting microglial M1/M2 polarization. J. Neuroinflammation 17 (1), 47. 10.1186/s12974-020-1726-7 32019561 PMC7001326

[B73] Liu W. Tang P. Wang J. Ye W. Ge X. Rong Y. (2021). Extracellular vesicles derived from melatonin-preconditioned mesenchymal stem cells containing USP29 repair traumatic spinal cord injury by stabilizing NRF2. J. Pineal Res. 71 (4), e12769. 10.1111/jpi.12769 34562326

[B74] Liu D. Shen H. Shen Y. Long G. He X. Zhao Y. (2021). Dual-cues laden scaffold facilitates neurovascular regeneration and motor functional recovery after complete spinal cord injury. Adv. Healthc. Mater 10 (10), e2100089. 10.1002/adhm.202100089 33739626

[B75] Liu X. Song S. Chen Z. Gao C. Li Y. Luo Y. (2022a). Release of O-GlcNAc transferase inhibitor promotes neuronal differentiation of neural stem cells in 3D bioprinted supramolecular hydrogel scaffold for spinal cord injury repair. Acta Biomater. 151, 148–162. 10.1016/j.actbio.2022.08.031 36002129

[B76] Liu X. Zhang L. Xu Z. Xiong X. Yu Y. Wu H. (2022b). A functionalized collagen-I scaffold delivers microRNA 21-loaded exosomes for spinal cord injury repair. Acta Biomater. 154, 385–400. 10.1016/j.actbio.2022.10.027 36270583

[B77] Liu Y. Wang Y. Wang Y. Zhou J. Ding W. (2023). The growth status and functions of olfactory ensheathing cells cultured on randomly oriented and aligned type-I-collagen-based nanofibrous scaffolds. Nanotechnology 35 (3). 10.1088/1361-6528/ad02a4 37905427

[B78] Liu G. Pei Z. Bai H. Huo L. Deng B. Jiang S. (2024). Biomaterial-mediated delivery of traditional Chinese medicine ingredients for spinal cord injury: a systematic review. Front. Pharmacol. 15, 1461708. 10.3389/fphar.2024.1461708 39545067 PMC11560789

[B79] Liu Y. Lin F. Wu C. Liu W. Wang H. Xiao C. (2024). *In situ* reaction-generated aldehyde-scavenging polypeptides-curcumin conjugate nanoassemblies for combined treatment of spinal cord injury. ACS Nano 18 (10), 7346–7362. 10.1021/acsnano.3c08662 38416031

[B80] Liu D. Shen H. Zhang K. Shen Y. Wen R. He X. (2024). Functional hydrogel Co-Remolding migration and differentiation microenvironment for severe spinal cord injury repair. Adv. Healthc. Mater 13 (3), e2301662. 10.1002/adhm.202301662 37937326

[B81] Liu Z. Zhao J. Wen H. Qin Y. Zheng L. Sun Y. (2025). Engineered lesion-homing liposomes encapsulating rosuvastatin enhance spinal cord injury repair by preventing the formation of foamy macrophages. J. Control Release. 388 (Pt 1), 114308. 10.1016/j.jconrel.2025.114308 41077147

[B82] Liu D. Niu R. Wang S. Shao L. Yang X. Liu X. (2025). Nitric oxide-releasing mesoporous hollow cerium oxide nanozyme-based hydrogel synergizes with neural stem cell for spinal cord injury repair. ACS Nano 19 (2), 2591–2614. 10.1021/acsnano.4c14261 39723955

[B83] Lu D. Wu J. P. Yang Q. W. Wang H. Y. Zhang G. G. (2023). Recent advances in lipid nanovesicles for targeted treatment of spinal cord injury. Front. Bioeng. Biotechnol. 11, 1261288. 10.3389/fbioe.2023.1261288 PMC10486273 37691909

[B84] M Alsharbaty M. H. Alazragi R. S. Mekaael Y. S. Hussein U. A. R. Mudhafar M. Alsailawi H. A. (2026). Nanoformulated fingolimod attenuates NLRP3 inflammasome activation and promotes functional recovery in a rat model of spinal cord injury. Spinal Cord. 64 (8), 669–677. 10.1038/s41393-026-01177-2 41723253

[B85] Ma D. Zhao Y. Huang L. Xiao Z. Chen B. Shi Y. (2020). A novel hydrogel-based treatment for complete transection spinal cord injury repair is driven by microglia/macrophages repopulation. Biomaterials 237, 119830. 10.1016/j.biomaterials.2020.119830 32036301

[B86] Ma D. Shen H. Chen F. Liu W. Zhao Y. Xiao Z. (2022). Inflammatory microenvironment-responsive nanomaterials promote spinal cord injury repair by targeting IRF5. Adv. Healthc. Mater 11 (23), e2201319. 10.1002/adhm.202201319 36165212

[B87] Ma Y. Pan J. Ju C. Yu X. Wang Y. Li R. (2025). Antioxidant nanozymes: current status and future perspectives in spinal cord injury treatments. Theranostics 15 (13), 6146–6183. 10.7150/thno.114836 40521206 PMC12159832

[B88] Ma Y. Ju C. Yu X. Zhao Y. Liu S. Li H. (2026). Multifunctional diselenide-based antioxidative nanozymes promote spinal cord repair *via* oxidative stress alleviation and immune modulation. ACS Nano 20 (5), 4116–4142. 10.1021/acsnano.5c15784 41582499

[B89] Mou C. Xia Z. Wang X. Dai X. Wang J. Zhang C. (2025). Stem cell-derived exosome treatment for acute spinal cord injury: a systematic review and meta-analysis based on preclinical evidence. Front. Neurol. 16, 1447414. 10.3389/fneur.2025.1447414 PMC11802430 39926016

[B90] Mousavi S. R. Rafati A. Dehghanian A. R. Nejat H. Matin F. Ghaffari M. K. (2022). The comparative effects of dexamethasone, nanocurcumin, and coenzyme Q10 against lumbar laminectomy-induced epidural fibrosis in a rat model. World Neurosurg. 167, e317–e322. 10.1016/j.wneu.2022.08.016 35963607

[B91] Mozhdehbakhsh Mofrad Y. Shamloo A. (2023). The effect of conductive aligned fibers in an injectable hydrogel on nerve tissue regeneration. Int. J. Pharm. 645, 123419. 10.1016/j.ijpharm.2023.123419 37717716

[B92] Nejati-Koshki K. Mortazavi Y. Pilehvar-Soltanahmadi Y. Sheoran S. Zarghami N. (2017). An update on application of nanotechnology and stem cells in spinal cord injury regeneration. Biomed. Pharmacother. 90, 85–92. 10.1016/j.biopha.2017.03.035 28343075

[B93] Nikolai R. J. Donsante A. Lamanna J. J. Cadena M. Jhunjhunwala A. Qin D. (2026). Intrathecal delivery of macromolecules to the spinal cord enabled by laser-activated perfluorocarbon nanodroplets. ACS Nano Med. 1 (6), 1447–1455. 10.1021/acsnanomed.5c00207 42433262 PMC13352938

[B94] Niu S. Liu X. Xu R. Zhu A. Zhu S. Hu F. (2026). Multifunctional nanozyme platforms in central nervous system therapies: from rational design to translational medicine. Theranostics 16 (14), 8385–8426. 10.7150/thno.136080 42559376 PMC13440536

[B95] Puhl D. L. Funnell J. L. Nelson D. W. Gottipati M. K. Gilbert R. J. (2020). Electrospun fiber scaffolds for engineering glial cell behavior to promote neural regeneration. Bioeng. (Basel) 8 (1), 4. 10.3390/bioengineering8010004 PMC7823609 33383759

[B96] Qian L. Yang K. Liu X. Zhang L. Zhao H. Qiu L. Z. (2023). Baicalein-functionalized collagen scaffolds direct neuronal differentiation toward enhancing spinal cord injury repair. Biomater. Sci. 11 (2), 678–689. 10.1039/d2bm01467j 36511438

[B97] Qin Q. Jin B. Bai C. Zhou Z. Shan B. Ding C. (2025). Biomimetic membrane-coating zinc sulfide nanoparticles for anti-inflammatory combined neuroprotective therapy for spinal cord injury. Bioact. Mater 56, 217–231. 10.1016/j.bioactmat.2025.09.032 41146852 PMC12554133

[B98] Salvi B. V. Kantak M. Kharangate K. Trotta F. Maher T. Shende P. (2024). Blind spots in development of nanomedicines. Technol. Cancer Res. Treat. 23, 15330338241245342. 10.1177/15330338241245342 38567427 PMC10993670

[B99] Santi S. Corridori I. Pugno N. M. Motta A. Migliaresi C. (2021). Injectable scaffold-systems for the regeneration of spinal cord: advances of the past decade. ACS Biomater. Sci. Eng. 7 (3), 983–999. 10.1021/acsbiomaterials.0c01779 33523634

[B100] Shen H. Wu S. Chen X. Xu B. Ma D. Zhao Y. (2020). Allotransplantation of adult spinal cord tissues after complete transected spinal cord injury: long-term survival and functional recovery in canines. Sci. China Life Sci. 63 (12), 1879–1886. 10.1007/s11427-019-1623-5 32382980

[B101] Shen K. Sun G. Chan L. He L. Li X. Yang S. (2021). Anti-inflammatory nanotherapeutics by targeting matrix metalloproteinases for immunotherapy of spinal cord injury. Small 17 (41), e2102102. 10.1002/smll.202102102 34510724

[B102] Shi J. Feng Y. Zhang S. Shao W. Ren N. Wang J. (2026). Intracellular small molecule/ion storm from Ca-phytate nanoparticle synergizes neural stem cell therapy and immunomodulation in spinal cord injury. Biomaterials 326, 123708. 10.1016/j.biomaterials.2025.123708 40992158

[B103] Singh N. Guha L. Pathak Z. Kumar H. (2026). Nano-shields: exploring the role of antioxidant mimicking nanoparticles as regenerative therapy in spinal cord injury. Biomater. Adv. 178, 214484. 10.1016/j.bioadv.2025.214484 40911982

[B104] Song S. Li Y. Huang J. Cheng S. Zhang Z. (2023). Inhibited astrocytic differentiation in neural stem cell-laden 3D bioprinted conductive composite hydrogel scaffolds for repair of spinal cord injury. Biomater. Adv. 148, 213385. 10.1016/j.bioadv.2023.213385 36934714

[B105] Su H. Chen Y. Tang B. Xiao F. Sun Y. Chen J. (2024). Natural and bio-engineered stem cell-derived extracellular vesicles for spinal cord injury repair: a meta-analysis with trial sequential analysis. Neuroscience 562, 135–147. 10.1016/j.neuroscience.2024.10.018 39490519

[B106] Sugeno A. Piao W. Yamazaki M. Takahashi K. Arikawa K. Matsunaga H. (2021). Cortical transcriptome analysis after spinal cord injury reveals the regenerative mechanism of central nervous system in CRMP2 knock-in mice. Neural Regen. Res. 16 (7), 1258–1265. 10.4103/1673-5374.301035 33318403 PMC8284262

[B107] Sun X. Xiong T. Yang K. Wang L. Yang W. Zhao H. (2023). Individually tailored modular “Egg” hydrogels capable of spatiotemporally controlled drug release for spinal cord injury repair. Adv. Healthc. Mater 12 (27), e2301169. 10.1002/adhm.202301169 37405810

[B108] Sun X. Yu J. Deng W. Xu X. (2026). Nano-primed reprogrammed neurons suppress the glial scar and facilitate axonal regeneration after spinal cord injury. J. Mater Chem. B 14 (21), 6606–6623. 10.1039/d6tb00620e 42132707

[B109] Sun M. L. Wu H. Liu S. Y. Jiang C. L. Zhou H. Hou A. (2026). Mesenchymal stem cell-lysate sustained-release nano-hydrogel alleviates spinal cord injury by inhibiting ferroptosis and mitochondrial intrinsic apoptosis. Int. Dent. J. 76 (3), 109557. 10.1016/j.identj.2026.109557 42001866 PMC13094645

[B110] Tian Z. Li H. Jiang Y. Wei H. Lu Y. Yao S. (2026). Dual-targeting nanoparticles enhance microglial P2Y12R expression to promote neuronal mitophagy for repairing spinal cord injury. Cell. Death Dis. 17 (1), 516. 10.1038/s41419-026-08596-2 42002569 PMC13221473

[B111] Usmani S. Franceschi Biagioni A. Medelin M. Scaini D. Casani R. Aurand E. R. (2020). Functional rewiring across spinal injuries *via* biomimetic nanofiber scaffolds. Proc. Natl. Acad. Sci. U. S. A. 117 (41), 25212–25218. 10.1073/pnas.2005708117 32999065 PMC7568334

[B112] Wan Y. Lin Y. Tan X. Gong L. Lei F. Wang C. (2024). Injectable hydrogel to deliver bone mesenchymal stem cells preloaded with azithromycin to promote spinal cord repair. ACS Nano 18 (12), 8934–8951. 10.1021/acsnano.3c12402 38483284

[B113] Wang X. J. Peng C. H. Zhang S. Xu X. L. Shu G. F. Qi J. (2019). Polysialic-acid-based micelles promote neural regeneration in spinal cord injury therapy. Nano Lett. 19 (2), 829–838. 10.1021/acs.nanolett.8b04020 30605619

[B114] Wang H. Lin F. Wu Y. Guo W. Chen X. Xiao C. (2023). Carrier-free nanodrug based on Co-Assembly of methylprednisolone dimer and rutin for combined treatment of spinal cord injury. ACS Nano 17 (13), 12176–12187. 10.1021/acsnano.3c00360 37387550

[B115] Wang W. Lu G. Guo P. Zhang H. Wang Y. Zheng D. (2025). Selenized neural stem cell-derived exosomes: a neotype therapeutic agent for traumatic injuries of the central nervous system. Cell. Rep. Med. 6 (9), 102319. 10.1016/j.xcrm.2025.102319 40882623 PMC12490213

[B116] Wang W. Liu E. Wang J. Li J. Lin S. Mei X. (2026). A novel schisantherin B-loaded Prussian blue nanozyme for treating spinal cord injury. J. Mater Chem. B 14 (10), 3187–3201. 10.1039/d5tb02205c 41479358

[B117] Waris A. Ali A. Khan A. U. Asim M. Zamel D. Fatima K. (2022). Applications of various types of nanomaterials for the treatment of neurological disorders. Nanomater. (Basel) 12 (13), 2140. 10.3390/nano12132140 PMC9268720 35807977

[B118] Wei R. Chen Y. Yang Q. Wang T. He Y. Yin N. (2025). Nanoenzyme-anchored mitofactories boost mitochondrial transplantation to restore locomotor function after paralysis following spinal cord injury. ACS Nano 19 (4), 4403–4421. 10.1021/acsnano.4c12557 39853984

[B119] Weidner N. Abel R. Maier D. Röhl K. Röhrich F. Baumberger M. (2025). Safety and efficacy of intrathecal antibodies to Nogo-A in patients with acute cervical spinal cord injury: a randomised, double-blind, multicentre, placebo-controlled, phase 2b trial. Lancet Neurol. 24 (1), 42–53. 10.1016/S1474-4422(24)00447-2 39706632

[B120] Wu W. Jia S. Xu H. Gao Z. Wang Z. Lu B. (2023). Supramolecular hydrogel microspheres of platelet-derived growth factor mimetic peptide promote recovery from spinal cord injury. ACS Nano 17 (4), 3818–3837. 10.1021/acsnano.2c12017 36787636

[B121] Wu H. Xing C. Yu B. Guo L. Dou X. Gao L. (2025). Metabolic reprogramming of neural stem cells by chiral nanofiber for spinal cord injury. ACS Nano 19 (4), 4785–4801. 10.1021/acsnano.4c15770 39841801 PMC11803919

[B122] Xia M. Zhao T. Wang X. Li Y. Li Y. Zheng T. (2021). Brain-derived neurotrophic factor and its applications through nanosystem delivery. Iran. J. Pharm. Res. 20 (4), 137–151. 10.22037/ijpr.2021.115705.15484 35194435 PMC8842625

[B123] Xiao Y. He Y. Zhong D. Liu B. Tang Z. Lan X. (2025). Effect of engineered cyanobacterial capsules on a neurogenic bladder after spinal cord injury. ACS Nano 19 (12), 11841–11860. 10.1021/acsnano.4c14140 40116782

[B124] Xin N. Liu X. Chen S. Zhang Y. Wei D. Sun J. (2022). Neuroinduction and neuroprotection co-enhanced spinal cord injury repair based on IL-4@ZIF-8-loaded hyaluronan-collagen hydrogels with nano-aligned and viscoelastic cues. J. Mater Chem. B 10 (33), 6315–6327. 10.1039/d2tb01111e 35920356

[B125] Xiong W. Liu M. Wang J. Liu J. Zheng M. Chan P. (2025a). Multidimensional engineering of extracellular vesicles for targeted delivery and microglial reprograming in spinal cord injury repair. ACS Nano 19 (35), 31551–31571. 10.1021/acsnano.5c08573 PMC12424966 40845835

[B126] Xiong T. Xiao X. Zhao H. Yang W. Gao X. Yang K. (2025b). The key to spinal cord recovery: harnessing p21 inhibition to boost neural stem/progenitor cell proliferation. ACS Nano 19 (30), 27406–27423. 10.1021/acsnano.5c05512 40712080

[B127] Xu Z. X. Zhang L. Q. Wang C. S. Chen R. S. Li G. S. Guo Y. (2017a). Acellular spinal cord scaffold implantation promotes vascular remodeling with sustained delivery of VEGF in a rat spinal cord hemisection model. Curr. Neurovasc Res. 14 (3), 274–289. 10.2174/1567202614666170718093508 28721809

[B128] Xu B. Zhao Y. Xiao Z. Wang B. Liang H. Li X. (2017b). A dual functional scaffold tethered with EGFR antibody promotes neural stem cell retention and neuronal differentiation for spinal cord injury repair. Adv. Healthc. Mater 6, 1601279. 10.1002/adhm.201601279 28233428

[B129] Xu B. Liu D. Liu W. Long G. Liu W. Wu Y. (2023). Engineered human spinal cord-like tissues with dorsal and ventral neuronal progenitors for spinal cord injury repair in rats and monkeys. Bioact. Mater 27, 125–137. 10.1016/j.bioactmat.2023.03.015 37064803 PMC10090126

[B130] Xu Z. Liu X. Pang Y. Chen Z. Jiang Y. Liu T. (2024). Long-acting heterodimeric paclitaxel-idebenone prodrug-based nanomedicine promotes functional recovery after spinal cord injury. Nano Lett. 24 (11), 3548–3556. 10.1021/acs.nanolett.4c00856 38457277

[B131] Xue W. Zhao Y. Xiao Z. Wu X. Ma D. Han J. (2020). Epidermal growth factor receptor-extracellular-regulated kinase blockade upregulates TRIM32 signaling Cascade and promotes neurogenesis after spinal cord injury. Stem Cells 38 (1), 118–133. 10.1002/stem.3097 31621984

[B132] Xue W. Fan C. Chen B. Zhao Y. Xiao Z. Dai J. (2021a). Direct neuronal differentiation of neural stem cells for spinal cord injury repair. Stem Cells 39 (8), 1025–1032. 10.1002/stem.3366 33657255

[B133] Xue W Zhang H. (#) Fan Y. Xiao Z. Zhao Y. Liu W. (2021b). Upregulation of Apol8 by epothilone D facilitates the neuronal relay of transplanted NSCs in spinal cord injury. Stem Cell. Res. Ther. 12 (1), 300. 10.1186/s13287-021-02375-w PMC8157417 34039405

[B134] Yamane K. Mazaki T. Shiozaki Y. Yoshida A. Shinohara K. Nakamura M. (2018). Collagen-binding hepatocyte growth factor (HGF) alone or with a Gelatin-furfurylamine hydrogel enhances functional recovery in mice after spinal cord injury. Sci. Rep. 8 (1), 917. 10.1038/s41598-018-19316-y 29343699 PMC5772669

[B135] Yamane K. Misawa H. Takigawa T. Ito Y. Ozaki T. Matsukawa A. (2019). Multipotent neurotrophic effects of hepatocyte growth factor in spinal cord injury. Int. J. Mol. Sci. 20 (23), 6078. 10.3390/ijms20236078 31810304 PMC6928986

[B136] Yang D. Yang W. Li L. Zhou K. Hao M. Feng X. (2019). Highly sensitive microstructure-based flexible pressure sensor for quantitative evaluation of motor function recovery after spinal cord injury. Sensors (Basel) 19 (21), 4673. 10.3390/s19214673 31661821 PMC6864470

[B137] Yang W. Li K. Pan Q. Huang W. Xiao Y. Lin H. (2024). An engineered bionic nanoparticle sponge as a cytokine trap and reactive oxygen species scavenger to relieve disc degeneration and discogenic pain. ACS Nano 18 (4), 3053–3072. 10.1021/acsnano.3c08097 38237054 PMC10832058

[B138] Yang S. Xue B. Zhang Y. Wu H. Yu B. Li S. (2025). Engineered extracellular vesicles from antler blastema progenitor cells: a therapeutic choice for spinal cord injury. ACS Nano 19 (6), 5995–6013. 10.1021/acsnano.4c10298 PMC11841045 39841785

[B139] Yao Y. Dong X. Pang Z. Shao J. He Z. Liu K. (2025). A zinc-citrate metal-organic framework-based adaptable hydrogen sulfide delivery System for regulating neuroregeneration microenvironment in spinal cord injury. ACS Nano 19 (25), 22798–22819. 10.1021/acsnano.4c18918 40524455

[B140] Yari-Ilkhchi A. Mahkam M. Ebrahimi-Kalan A. Zangbar H. S. (2024). Design and synthesis of nano-biomaterials based on graphene and local delivery of cerebrolysin into the injured spinal cord of mice, promising neural restoration. Nanoscale Adv. 6 (3), 990–1000. 10.1039/d3na00760j 38298594 PMC10825937

[B141] Ying Y. Cai X. Dai P. Zhang Y. Lv J. Huang Z. (2025). Neurological emergency treatment strategy: a neuron-targeted regulation System for reactive oxygen species metabolism through ferroptosis modulation. ACS Nano 19 (9), 8753–8772. 10.1021/acsnano.4c15705 39996314 PMC11913020

[B142] You Z. Gao X. Kang X. Yang W. Xiong T. Li Y. (2023). Microvascular endothelial cells derived from spinal cord promote spinal cord injury repair. Bioact. Mater 29, 36–49. 10.1016/j.bioactmat.2023.06.019 PMC10444976 37621772

[B143] Yuan T. Wang T. Zhang J. Liu P. Xu J. Gu Z. (2023). Robust and multifunctional nanoparticles assembled from natural polyphenols and metformin for efficient spinal cord regeneration. ACS Nano 17 (18), 18562–18575. 10.1021/acsnano.3c06991 37708443

[B144] Zhang H. Sha J. Cao S. Feng J. Pang X. Xu D. (2025). Engineering neural recovery: Micro/Nano-structured materials for nerve regeneration. Mater Today Bio 35, 102538. 10.1016/j.mtbio.2025.102538 41322153 PMC12661459

[B145] Zhai X. Chen K. Yang H. Li B. Zhou T. Wang H. (2021). Extracellular vesicles derived from CD73 modified human umbilical cord mesenchymal stem cells ameliorate inflammation after spinal cord injury. J. Nanobiotechnology 19 (1), 274. 10.1186/s12951-021-01022-z 34496892 PMC8425042

[B146] Zhang H. Xue W. Xue X. Fan Y. Yang Y. Zhao Y. (2021). Spatiotemporal dynamic changes, proliferation, and differentiation characteristics of Sox9-positive cells after severe complete transection spinal cord injury. Exp. Neurol. 337, 113556. 10.1016/j.expneurol.2020.113556 33326799

[B147] Zhang L. Fan C. Hao W. Zhuang Y. Liu X. Zhao Y. (2021). NSCs migration promoted and drug delivered exosomes-collagen scaffold *via* a bio-specific peptide for one-step spinal cord injury repair. Adv. Healthc. Mater 10 (8), e2001896. 10.1002/adhm.202001896 33522126

[B148] Zhang C. Wang A. Zhang G. Wu C. Rong W. Huo X. (2022). Experimental study of electric field stimulation combined with polyethylene glycol in the treatment of spinal cord injury in rats. Sheng Wu Yi Xue Gong Cheng Xue Za Zhi 39 (1), 10–18. 10.7507/1001-5515.202011035 PMC9927733 35231961

[B149] Zhang K. Wen R. Ma W. He X. Yang Z. Liu D. (2025). Neuronal dual-specificity phosphatase 26 inhibition *via* reactive-oxygen-species responsive mesoporous-silica-loaded hydrogel for spinal cord injury repair. ACS Nano 19 (4), 4942–4958. 10.1021/acsnano.4c16875 39846381

[B150] Zhang W. Hu Q. Li W. Zhou X. Liu M. Lu J. (2026). Spatiotemporal liposome-zwitterionic nano-hydrogel orchestrates immunomodulation and neurogenesis in spinal cord injury repair. Mater Today Bio 38, 103100. 10.1016/j.mtbio.2026.103100 42011490 PMC13092031

[B151] Zhang Z. Li H. Liu J. Shen N. Zhu Z. Qi X. (2026). Intraneural delivery of CBD3A6K-RhB *via* PEG-PLGA nanoparticles for neuropathic pain therapy. J. Control Release 392, 114662. 10.1016/j.jconrel.2026.114662 41620030

[B152] Zhao X. Gu R. Zhao Y. Wei F. Gao X. Zhuang Y. (2023). Adult spinal cord tissue transplantation combined with local tacrolimus sustained-release collagen hydrogel promotes complete spinal cord injury repair. Cell. Prolif. 56 (5), e13451. 10.1111/cpr.13451 36916024 PMC10212717

[B153] Zhao H. Xiong T. Chu Y. Hao W. Zhao T. Sun X. (2024). Biomimetic dual-network collagen fibers with porous and mechanical cues reconstruct neural stem cell niche *via* AKT/YAP mechanotransduction after spinal cord injury. Small 20 (32), e2311456. 10.1002/smll.202311456 38497893

[B154] Zhao S. Duan L. Lv Z. Han Z. Xu W. Zhang K. (2026). Extracellular vesicle-mediated precision delivery of paclitaxel activates mitophagy to promote repair after spinal cord injury. ACS Nano 20 (11), 9387–9406. 10.1021/acsnano.5c21273 41773788

[B155] Zheng J. Chen T. Wang K. Peng C. Zhao M. Xie Q. (2024). Engineered multifunctional zinc-organic framework-based aggregation-induced emission nanozyme for accelerating spinal cord injury recovery. ACS Nano 18 (3), 2355–2369. 10.1021/acsnano.3c10541 38197586

[B156] Zhou Z. Li C. Bao T. Zhao X. Xiong W. Luo C. (2022). Exosome-shuttled miR-672-5p from anti-inflammatory microglia repair traumatic spinal cord injury by inhibiting AIM2/ASC/Caspase-1 signaling pathway mediated neuronal pyroptosis. J. Neurotrauma. 39 (15-16), 1057–1074. 10.1089/neu.2021.0464 35243913

[B157] Zhou K. Wei W. Yang D. Zhang H. Yang W. Zhang Y. (2024). Dual electrical stimulation at spinal-muscular interface reconstructs spinal sensorimotor circuits after spinal cord injury. Nat. Commun. 15 (1), 619. 10.1038/s41467-024-44898-9 38242904 PMC10799086

[B158] Zhu R. Zhu X. Zhu Y. Wang Z. He X. Wu Z. (2021). Immunomodulatory layered double hydroxide nanoparticles enable neurogenesis by targeting transforming growth Factor-β receptor 2. ACS Nano 15 (2), 2812–2830. 10.1021/acsnano.0c08727 33527830

[B159] Zhu B. Gu G. Ren J. Song X. Li J. Wang C. (2023). Schwann cell-derived exosomes and methylprednisolone composite patch for spinal cord injury repair. ACS Nano 17 (22), 22928–22943. 10.1021/acsnano.3c08046 37948097

[B160] Zhuang Z. Li B. Cai C. Jiang Y. Tang J. Rong L. (2025). MMP-Responsive nanodrug loaded with glibenclamide for targeted repair of acute spinal cord injury. Int. J. Pharm. 675, 125526. 10.1016/j.ijpharm.2025.125526 40189167

[B161] Zou Y. Zhao Y. Xiao Z. Chen B. Ma D. Shen H. (2020). Comparison of regenerative effects of transplanting three-dimensional longitudinal scaffold loaded-human mesenchymal stem cells and human neural stem cells on spinal cord completely transected rats. ACS Biomater. Sci. Eng. 6 (3), 1671–1680. 10.1021/acsbiomaterials.9b01790 33455365

